# Pulmonary fibrosis after COVID-19 is characterized by airway abnormalities and elevated club cell secretory protein-16

**DOI:** 10.1172/jci.insight.199983

**Published:** 2026-07-08

**Authors:** Matthew R. Baldwin, Ansley E. Jones, David Zhang, Chandan Gurung, Zain Khan, Anjali Saqi, Xuehan Yang, Ying Wei, Renu Nandakumar, Scarlett O. Murphy, Claire F. McGroder, Faisal Shaikh, Selim Arcasoy, Luke Benvenuto, Harpreet Grewal, Benjamin M. Smith, Eric A. Hoffman, Agnes C.Y. Yuen, Parteek Johal, Christopher Carlsten, Christopher J. Ryerson, J. Brent Richards, Alyson W. Wong, Tomoko Nakanishi, Aditi S. Shah, Christine Kim Garcia

**Affiliations:** 1 Department of Medicine, Division of Pulmonary, Allergy, and Critical Care, Columbia University Vagelos College of Physicians and Surgeons, New York, New York, USA.; 2 Irving Institute for Clinical and Translational Research, Columbia University Medical Center, New York, New York, USA.; 3 Department of Biostatistics, Columbia University Mailman School of Public Health, New York, New York, USA.; 4 Department of Pathology, Columbia University Vagelos College of Physicians and Surgeons, New York, New York, USA.; 5 Department of Medicine, McGill University, Montreal, Quebec, Canada.; 6 Department of Radiology, Internal Medicine, and Biomedical Engineering, University of Iowa, Iowa City, Iowa.; 7 Division of Respiratory Medicine, Department of Medicine, University of British Columbia, Vancouver, British Columbia, Canada.; 8 Departments of Medicine, Human Genetics, Epidemiology and Biostatistics, McGill University, Montréal, Quebec, Canada.; 9 Department of Twin Research, King’s College London, London, United Kingdom.; 10 Department of Human Genetics, and; 11 Lady Davis Institute, Jewish General Hospital, McGill University, Montréal, Quebec, Canada.; 12 Kyoto-McGill International Collaborative Program in Genomic Medicine, Graduate School of Medicine, Kyoto University, Kyoto, Japan.; 13 Department of Genome Informatics, Graduate School of Medicine, the University of Tokyo, Tokyo, Japan.; 14 Research Fellow, Japan Society for the Promotion of Science, Tokyo, Japan.; 15 Columbia Precision Medicine Initiative, Columbia University Vagelos College of Physicians and Surgeons, New York, New York, USA.

**Keywords:** Infectious disease, Pulmonology, Biomarkers, COVID-19, Fibrosis

## Abstract

**BACKGROUND:**

There are no known serum biomarkers that provide mechanistic insight or prognostic enrichment for post–COVID-19 pulmonary fibrosis.

**METHODS:**

We tested associations of serum biomarkers with radiographic fibrosis-like abnormalities (reticulation, traction bronchiectasis, or honeycombing) on thoracic computed tomography (CT) scans 4 months, 15 months, and 3 years after hospitalization in an American discovery cohort of severe-to-critical COVID-19 survivors, and externally validated findings in 2 Canadian cohorts of moderate-to-critical COVID-19 survivors. In the discovery cohort, we investigated the dose-response relationship of the biomarker with CT-derived airway-to-lung ratio. We performed single-cell RNA sequencing (scRNA-seq) of transbronchial lung biopsies from COVID-19 survivors obtained 3 years after COVID-19 hospitalization and conducted immunofluorescence analysis of COVID-19 lung explants.

**RESULTS:**

Among 150 discovery cohort participants, only higher levels of circulating club cell secretory protein-16 (CC16, encoded by the *SCGB1A1* gene) at hospital discharge, 4 months, 15 months, and 3 years were associated with thoracic CT fibrosis-like abnormalities in cross-sectional and longitudinal analyses. Higher CC16 levels were associated with thoracic CT fibrosis-like abnormalities in 2 validation cohorts (*n* = 56 and *n* = 37). CC16 levels were linearly associated with increased airway-to-lung ratio. scRNA-seq revealed increased proportions of epithelial cells expressing *SCGB1A1* and *SCGB1A1/MUC5B* in COVID-19 survivors with fibrosis. Immunofluorescence analysis of COVID-19 lung explants demonstrated increased numbers of SCGB1A1-expressing epithelial cells only in small (<100 μm) airways, with 3-fold more CC16/MUC5B-coexpressing cells in respiratory bronchioles..CONCLUSION. Higher CC16 levels are associated with CT fibrosis-like abnormalities for up to 3 years following moderate-to-critical COVID-19. Increased CC16 reflects dysregulated small airway epithelial progenitor cell remodeling and increased expansion of CC16^+^MUC5B^+^ epithelial cells in respiratory bronchioles after COVID-19.

**TRIAL REGISTRATION:**

Not applicable.

**FUNDING:**

Department of Defense, NIH, and Japan Society for the Promotion of Science for Young Scientists.

## Abstract 2

A blood protein identifies people with lasting lung scarring after COVID-19 and reveals ongoing airway cell changes that may drive fibrosis years after infection.

## Introduction

Some survivors of coronavirus disease 2019 (COVID-19) have residual abnormalities consisting of persistent reticulations and traction bronchiectasis that are similar to radiographic findings of idiopathic pulmonary fibrosis (IPF) (1). Genome-wide association studies similarly suggest a shared genetic etiology between COVID-19 and IPF (2, 3). In COVID-19 acute respiratory distress syndrome (ARDS), there is an accumulation of profibrotic macrophage populations that are also found in IPF (4), and a time-dependent shift from proinflammatory to profibrotic gene expression patterns (5, 6).

Several longitudinal cohort studies of adults hospitalized with COVID-19 suggest that 30%–75% have persistent radiographic patterns consisting predominantly of reticulations and traction bronchiectasis during the first year after acute SARS-CoV-2 infection (1, 7–14). Greater initial severity of illness and receipt of invasive mechanical ventilation are major clinical risk factors for fibrosis-like abnormalities after COVID-19 (7–9, 15, 16). Reassuringly, fibrosis-like abnormalities are usually associated with only mild reductions in diffusion capacity, and they are not consistently associated with restrictive or obstructive ventilatory defects, reduced exercise tolerance, or dyspnea (7, 9, 10, 13, 15). However, these findings may be falsely reassuring since pulmonary fibrosis is usually an insidious subclinical process that becomes symptomatic only after lung function has declined substantially (17). To date, there are no known postacute blood-based biomarkers that offer mechanistic insight or prognostic enrichment for post–COVID-19 residual abnormalities on thoracic imaging.

Biomarkers of inflammation, aging, endothelial activation, pulmonary epithelial function, fibrosis, and fibrinolysis are associated with COVID-19 acute lung injury (ALI), interstitial lung abnormalities (ILAs), and idiopathic pulmonary fibrosis (IPF) (18–21). Given the observed overlap in radiographic patterns and mechanisms of disease between COVID-19 ALI, ILAs, and IPF, we hypothesized that biomarkers from the aforementioned classes would be associated with fibrotic pulmonary radiographic patterns in moderate-to-critical COVID-19 survivors during first 3 years following acute COVID-19 illness. Accordingly, we assessed biomarkers from several Luminex multiplex panels that included these biomarker classes.

In a single-center New York City–based 3-year prospective longitudinal discovery cohort of adults hospitalized with severe and critical COVID-19 in 2020, we found that only higher levels of club cell secretory protein (CC16), encoded by the *SCGB1A1* gene, were consistently associated with an increased risk of fibrosis-like abnormalities in adjusted analyses. We then sought to externally validate the association of postacute plasma CC16 levels and fibrotic radiographic patterns in 2 independent Canadian cohorts of adult moderate-to-critical COVID-19 survivors.

We tested the association between serum CC16 and larger airway-to-lung ratio on thoracic CT scans at 15-month follow-up in our discovery cohort. To investigate the pulmonary source of elevated serum CC16 levels, we analyzed single-cell RNA sequencing (scRNA-seq) and immunofluorescent staining of lung tissue from COVID-19 survivors.

## Results

### Participant characteristics, thoracic imaging, and biomarkers.

**Figure 1:**
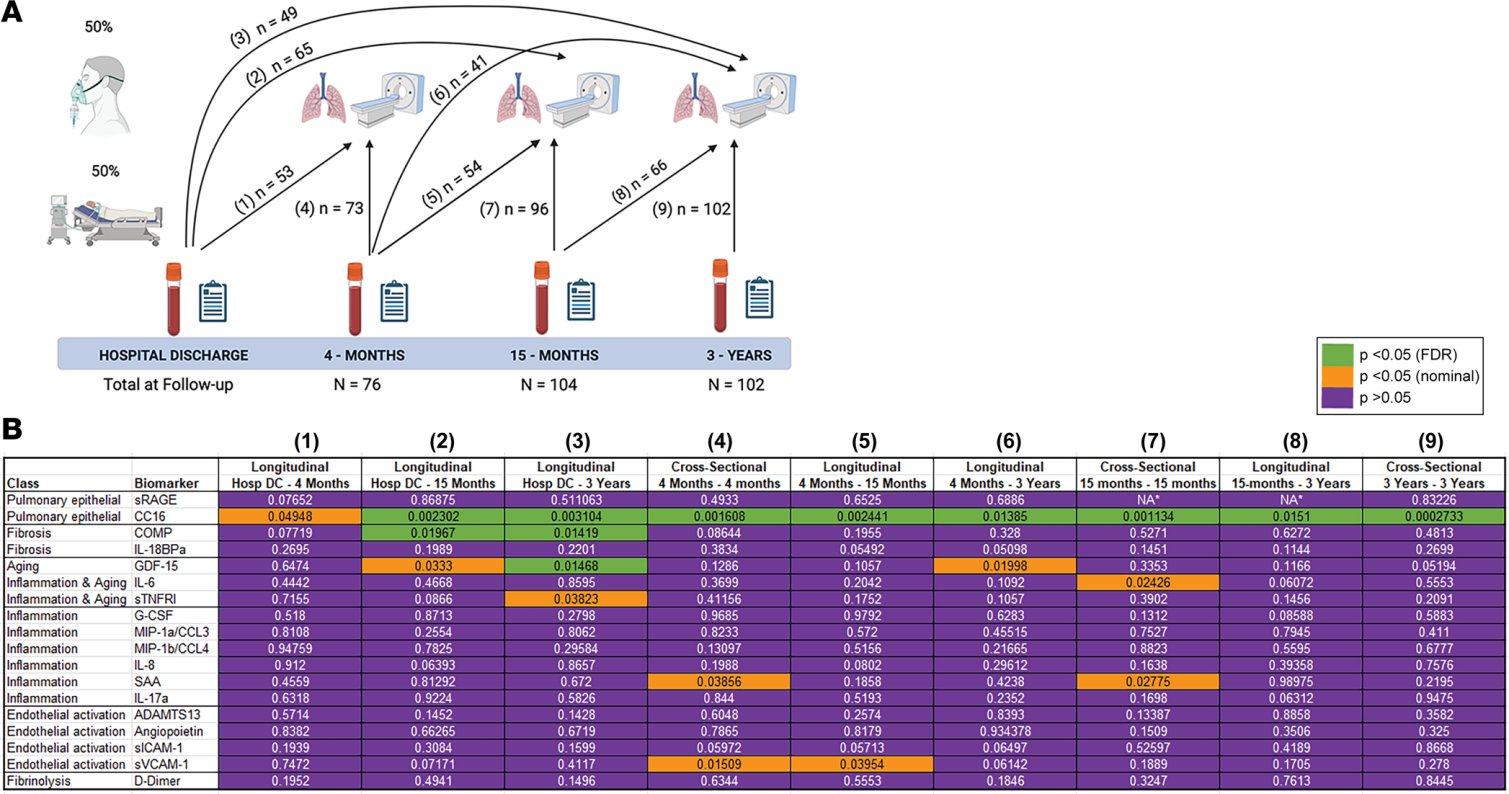
Discovery cohort study design and associations of biomarkers with fibrosis-like abnormalities. (**A**) Cohort study design of hospitalized survivors of severe and critical COVID-19, with sampling weighted for 50% invasive mechanical ventilation survivors. Schema of measurements and associations of blood biomarkers and high-resolution chest CT scans with sample size available at each time point. (**B**) Associations of serum biomarkers with fibrosis-like abnormalities. *P* values represent the *P*-for-association between the plasma biomarker and predictive risk of fibrotic pattern on thoracic CT using generalized additive models with LOESS smoothers, with adjustment for age, sex, race/ethnicity, BMI, COPD, asthma, pack-years history of smoking, estimated glomerular filtration rate, use of corticosteroids during COVID-19 hospitalization, IL-6 receptor inhibitor therapy during COVID-19 hospitalization, ventilator days, and days since initial SARS-CoV-2 infection utilizing covariate balanced propensity scores.

There were 150 adults in the Columbia University Irving Medical Center (hereafter, Columbia) discovery cohort with 76, 104, and 102 participants at 4-month, 15-month, and 3-year follow-up, respectively (Figure 1A). Forty-one (27%) participated in all 3 follow-up visits, 50 (33%) participated in 2 visits, and 59 (39%) participated in only 1 visit (Supplemental Figure 1; supplemental material available online with this article; https://doi.org/10.1172/jci.insight.199983DS1). Demographic and clinical characteristics did not appear to differ across follow-up visits (Supplemental Table 1) (13). There were 56 adults in the University of British Columbia (UBC) validation cohort with 3-month follow-up, and 37 adults in the McGill validation cohort. The Columbia cohort was predominantly Black or Hispanic, and the UBC and McGill validation cohorts were mostly White or Asian. Compared with Columbia participants, UBC and McGill participants were slightly older, less obese, and had a lower prevalence of asthma. Compared with the UBC and McGill cohorts, the Columbia cohort had greater severity of acute COVID-19 illness, with more patients receiving mechanical ventilation (46% vs. 21% and 5.4%) and having a longer median [IQR] hospital length of stay (18 [7–42] vs. 8.5 [5.0–13] and 7 [5.0–19] days) (Table 1).

The prevalence of fibrosis-like abnormalities at follow-up in the Columbia and UBC cohorts ranged from 57%–64%, and was 43% in the nested case-control cohort from McGill, with 16 participants with and 21 participants without fibrosis-like abnormalities, matching on demographics, comorbidities, and either hospital discharge, 4-month, or 15-month thoracic CT follow-up time. Fibrosis-like abnormalities were predominantly characterized by reticulations and traction bronchiectasis. We observed honeycombing in less than 5% of Columbia and UBC patients and in 4 (11%) McGill patients (Supplemental Tables 2 and 3).

**Figure 2:**
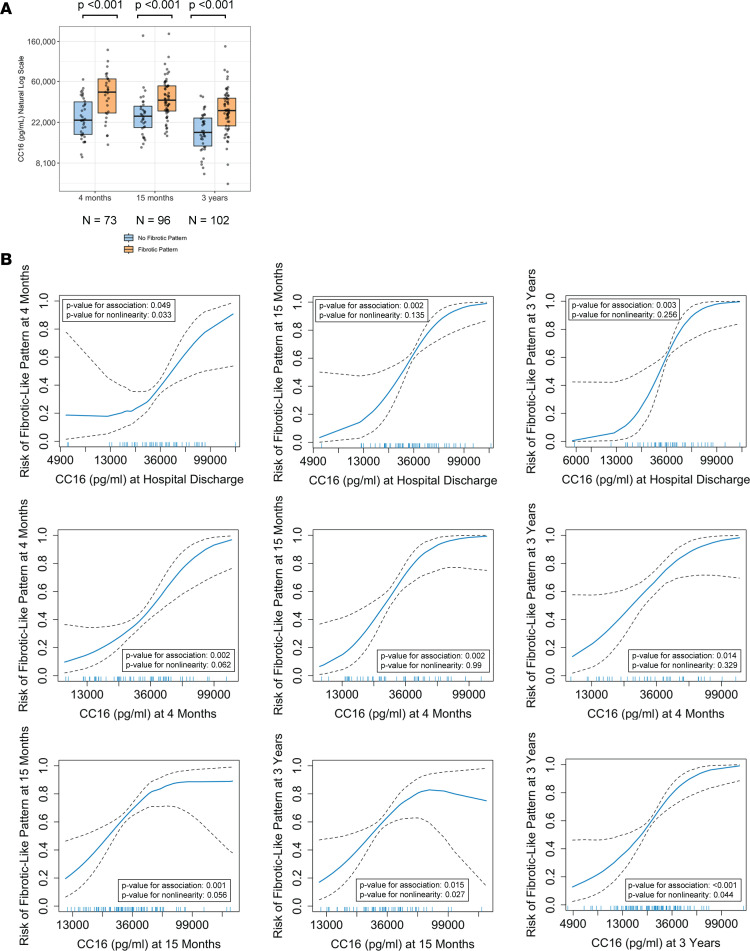
Associations of CC16 with fibrosis-like abnormalities in the discovery cohort. (**A**) Dot-box plots of plasma CC16 at hospital discharge, 4 months, 15 months, and 3 years, stratified by fibrotic pattern status in the Columbia (discovery) cohort. *P* values are derived from Mann-Whitney tests. Boxes represent the IQR, and the middle bars represent the median. (**B**) Plots of generalized additive models (GAM) with LOESS smoothers with adjustment for age, sex, race/ethnicity, BMI, COPD, asthma, pack-year history of smoking, estimated glomerular filtration rate, use of corticosteroids during COVID-19 hospitalization, IL-6 receptor inhibitor therapy during COVID-19 hospitalization, ventilator days, and days since initial SARS-CoV-2 infection using covariate balanced propensity scores.

In the Columbia cohort, of the 18 biomarkers tested, only CC16 had consistent significant associations with the predicted risk of fibrosis-like abnormalities in adjusted analyses over 3 years of follow-up (Figure 1B). Sensitivity analyses revealed potential model overfitting with high outliers after natural log transformation observed in 6 end-stage renal disease (ESRD) dialysis patients. Since CC16 is renally excreted (22, 23), we report CC16 analyses excluding Columbia participants with ESRD and excluded ESRD patients in validation cohorts. CC16 declined slightly over 3 years but remained consistently higher among those with fibrosis-like abnormalities (Figure 2A), including in exploratory longitudinal analyses (Supplemental Figure 2). In 9 cross-sectional and longitudinal adjusted associations tested between hospital discharge and 3-year follow-up, CC16 had consistent, large magnitude, direct, and mostly linear associations with the predicted risk of fibrosis-like abnormalities (Figure 2B). The adjusted odds of fibrosis-like abnormalities for every natural log fold increase in CC16 ranged from 5.0 (95% CI: 1.6–20.7) to 16 (95% CI: 4.1–111). Adjusted odds ratios (ORs) increased 2- to 7-fold across tertiles of CC16 (all *P*-for-trend < 0.02) (Supplemental Table 4). Higher CC16 had a moderate inverse correlation with lower percentage predicted diffusion capacity of carbon monoxide (DLCO) at 4 months and 3 years but had no statistically significant correlation with DLCO at 15 months (Supplemental Table 5). In post hoc unadjusted and adjusted cross-sectional analyses, CC16 did not demonstrate consistent inverse associations with telomere length, with an association only detected between CC16 and telomere length modeled as a continuous variable at 4-month follow-up (Supplemental Table 6).

**Figure 3:**
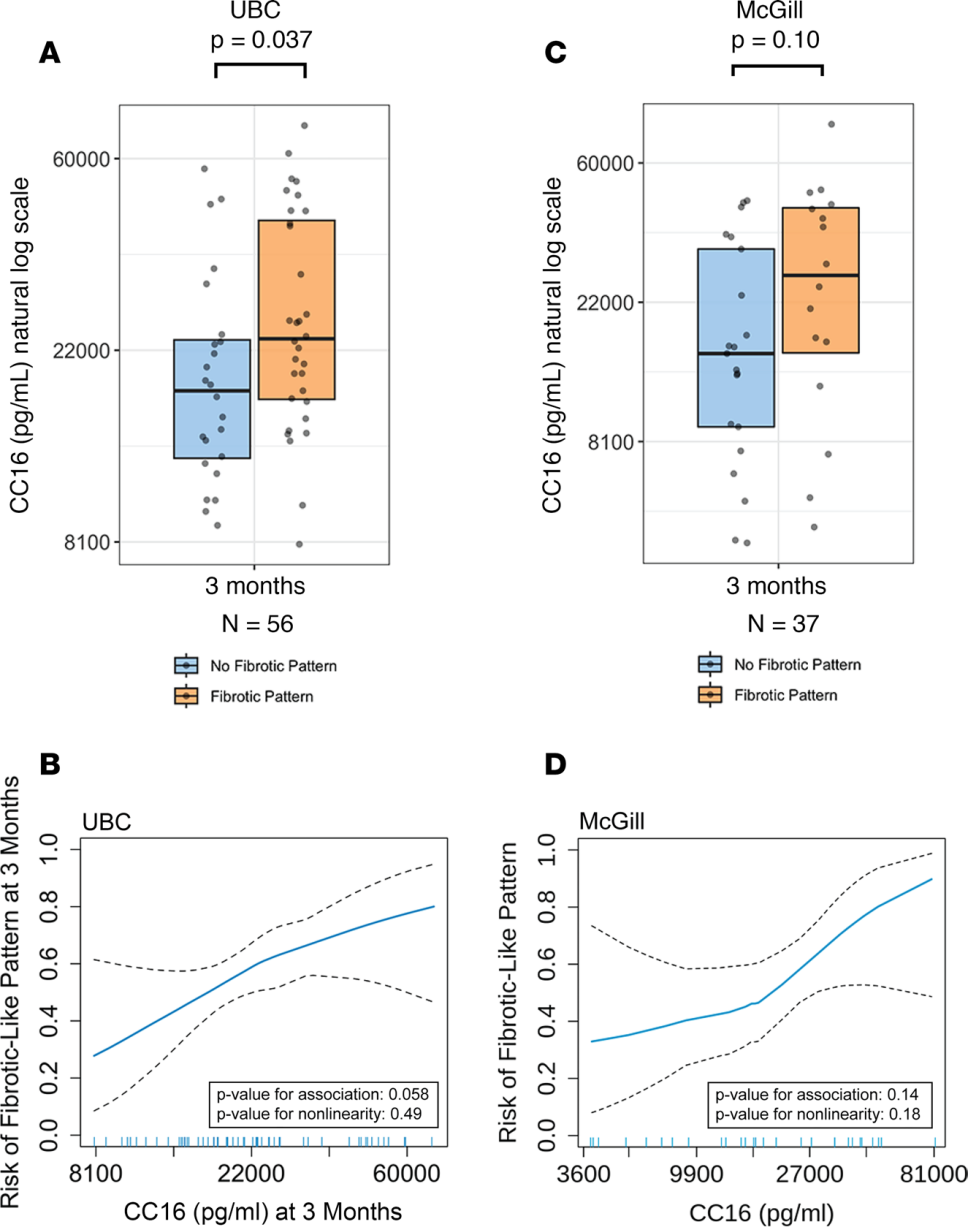
Associations of CC16 with fibrosis-like abnormalities in the validation cohorts. (**A** and **C**) Dox-box plots of plasma CC16 stratified by fibrotic pattern status in the University of British Columbia (UBC) and McGill University validation cohorts. Boxes represent the IQR and the middle bars represent the median. *P* values are derived from Mann-Whitney tests. (**B** and **D**) Plots of generalized additive models (GAM) with LOESS smoothers with adjustment for age, sex, race/ethnicity, BMI, COPD, asthma, pack-year history of smoking, estimated glomerular filtration rate, use of corticosteroids during COVID-19 hospitalization, IL-6 receptor inhibitor therapy during COVID-19 hospitalization, ventilator days, and days since initial SARS-CoV-2 infection using covariate balanced propensity scores.

In the UBC cohort, 3-month cross-sectional analyses revealed a direct, linear, borderline significant association between CC16 and the predicted risk of fibrosis-like abnormalities (generalized additive model [GAM] *P* = 0.058) that was statistically significant in unadjusted (*P* = 0.037) and adjusted logistic regression analyses (OR 2.92, 95% CI per natural log fold increase in CC16: 1.07–9.07) (Figure 3 and Supplemental Table 7). Higher CC16 had a moderate inverse correlation with lower percentage predicted DLCO (Supplemental Table 5).

Given the small sample size of the McGill cohort, we tested associations and estimated effect sizes of CC16 with fibrosis-like abnormalities for the hospital discharge, 4-month, and 15-month follow-up participants combined. We observed a similar direct, linear, but non-significant association in unadjusted and adjusted analyses (Figure 3, C and D), with an adjusted OR of fibrosis-like abnormalities of 2.36 (95% CI: 0.93–7.03) per natural log fold increase in CC16 (Supplemental Table 7). In unadjusted analyses stratified by follow-up time period, we observed nonsignificant higher median CC16 levels in those with fibrosis-like abnormalities (Supplemental Figure 3). In post hoc sensitivity analyses excluding 3 patients with possible honeycombing found on thoracic CT greater than 5 years prior to the COVID-19 pandemic, we observed the magnitude of the associations to be slightly less, with the data still collectively suggesting an association of higher CC16 levels with fibrosis-like abnormalities, even though the sample size becomes small in analyses stratified by follow-up time period (Supplemental Table 7 and Supplemental Figure 4).

**Figure 4:**
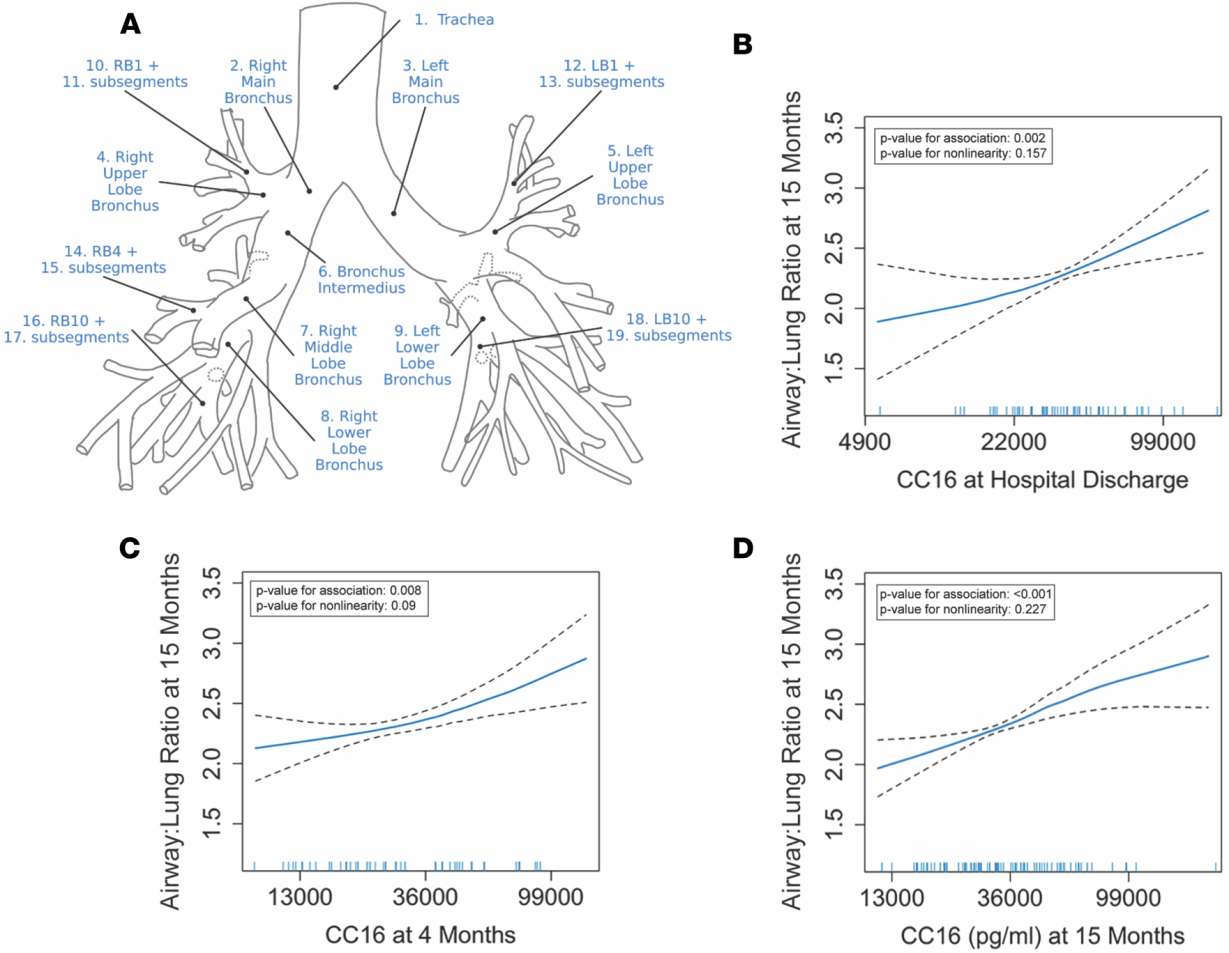
Associations of CC16 with airway-to-lung ratio measured on 15-month thoracic CT in the discovery cohort. (**A**) Using Apollo software (VIDA Diagnostics), airway-to-lung ratio was quantified on inspiratory thoracic CT performed at 15 months as the geometric mean of airway lumen diameters measured at 19 standard anatomic locations divided by the cube root of lung volume. (**B**–**D**) Plots are of generalized additive models (GAM) with LOESS smoothers with adjustment for age, sex, race/ethnicity, BMI, COPD, asthma, pack-year history of smoking, estimated glomerular filtration rate, use of corticosteroids during COVID-19 hospitalization, IL-6 receptor inhibitor therapy during COVID-19 hospitalization, ventilator days, and days since initial SARS-CoV-2 infection using covariate balanced propensity scores.

In radiographic analyses of the Columbia cohort, higher levels of CC16 were directly and linearly associated with a higher airway-to-lung ratio at 15 months in adjusted longitudinal and cross-sectional analyses (Figure 4), with consistent effect sizes over time. For example, every natural log fold increase in CC16 at hospital discharge was associated with a 0.61 (0.21–1.01) standard deviation (SD) unit increase airway-to-lung ratio (Supplemental Table 8). Associations were robust to complete-case and larger airway (presubsegmental) sensitivity analyses (Supplemental Figure 5).

### Functional analyses with lung tissue.

**Figure 5:**
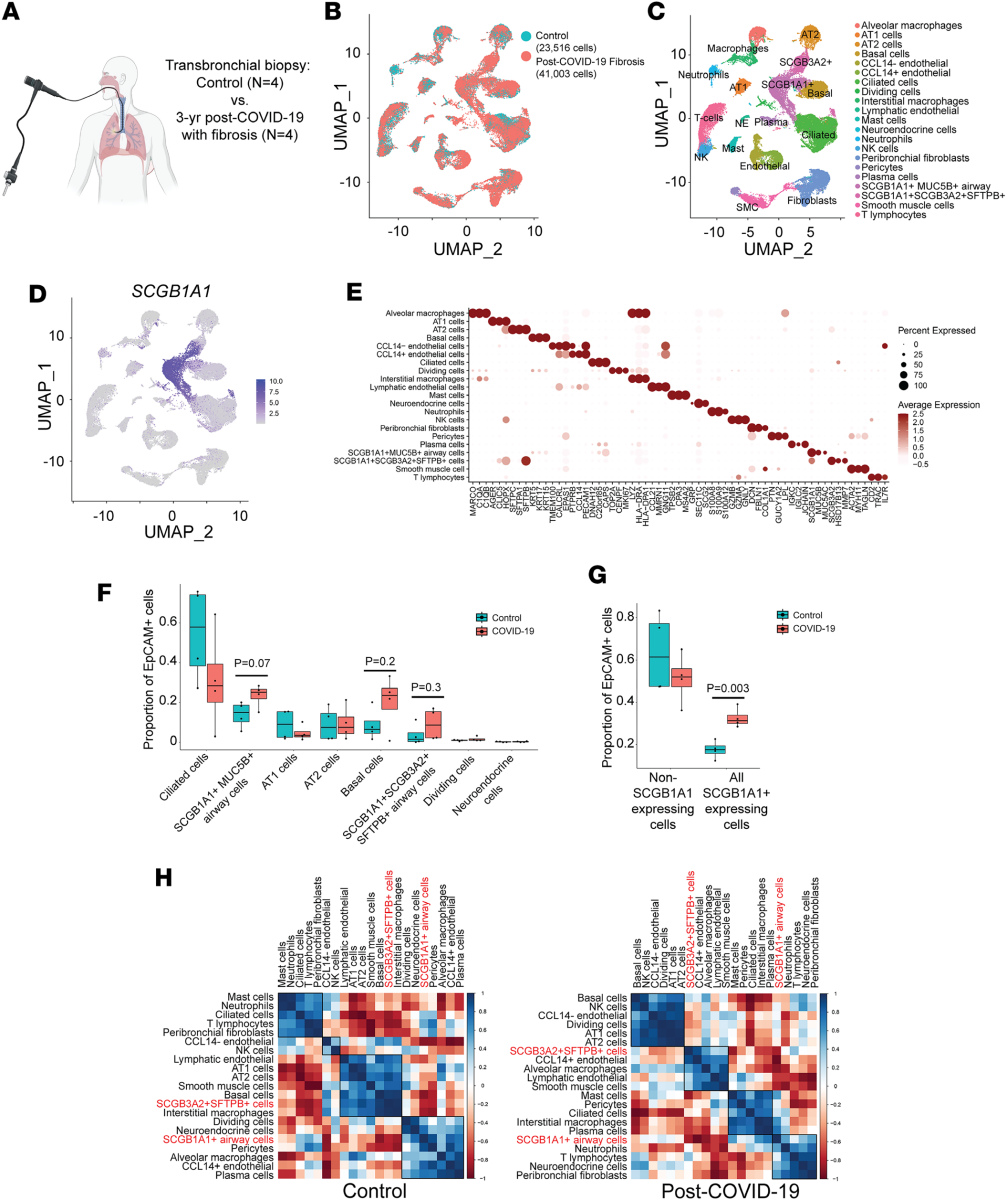
Increased proportions of SCGB1A1-expressing lung cells after severe COVID-19. (**A**) Schematic. (**B**) UMAP plot of cells from participants with control lung (aqua) and post–COVID-19 with fibrosis (red). (**C)** UMAP plot of annotated cell types. (**D**) Feature plot of *SCGB1A1* expression. (**E**) Dot blot plot of top expression genes in each of the different cell clusters. (**F**) Proportion of different subsets of lung epithelial (EpCAM^+^) cells found in control and COVID-19 with fibrosis. (**G**) Proportion of SCGB1A1-expressing cells (from the SCGB1A1^+^MUC5B^+^ airway and SCGB1A1^+^SCGB3A2^+^SFTPB^+^ airway cell clusters) and non–SCGB1A1-expressing cells (all other EpCAM^+^ cell clusters) found in controls and COVID-19 with fibrosis. (**H**) Proportions of SCGB1A1-expressing cells and other cell types in controls and COVID-19 with fibrosis samples. *P* values are from Mann-Whitney (**F**) or Fischer’s exact tests (**G**).

The demographics and clinical characteristics of 4 COVID-19 survivors with the highest fibrosis scores on thoracic imaging at 3-year follow-up (24) and 4 control participants (lung transplant recipients without histopathologic evidence of rejection or infection, Supplemental Figure 6) are described in Supplemental Table 9 (Figure 5A). A single-cell suspension of each transbronchial lung biopsy sample was sorted by fluorescence-activated cell sorting (FACS) to mix epithelial, immune/endothelial, and non-epithelial/non-immune/non-endothelial cells in a 1:1:1 ratio prior to sequencing (Figure 5A). Clustering and distinction of epithelial cell types by the expression of top genes shows 2 groups of cells expressing *SCGB1A1* (Figure 5, C–D). The first, designated as SCGB1A1^+^MUC5B^+^ airway cells, do not express surfactant genes but robustly express mucin genes; these are secretory cells (Figure 5E). The second, designated as SCGB1A1^+^SCGB3A2^+^SFTPB^+^ airway cells, are similar to the previously described preterminal bronchiole secretory cells (pre-TB-SC) or respiratory airways secretory (RAS) cells (Figure 5E and Supplemental Figure 7) (25, 26). There is evidence of increased proportions of both of these SCGB1A1-expressing cell clusters when analyzed separately (Figure 5F) or in combination (Figure 5G) in COVID-19 samples as compared with control samples. Proportions of SCGB1A1-expressing cell clusters were more dissimilar between COVID-19 and control samples than the proportions of alveolar type 1 and type 2 (AT1 and AT2) epithelial cells (Figure 5H), suggesting that post-COVID-19 effects drive expansion of airway rather than alveolar cells. Differences in fibroblast, immune, and endothelial populations between COVID-19 and control samples are shown in Supplemental Figure 7. None of the COVID-19 cases carried the MUC5B risk allele that has been linked to pulmonary fibrosis.

**Figure 6:**
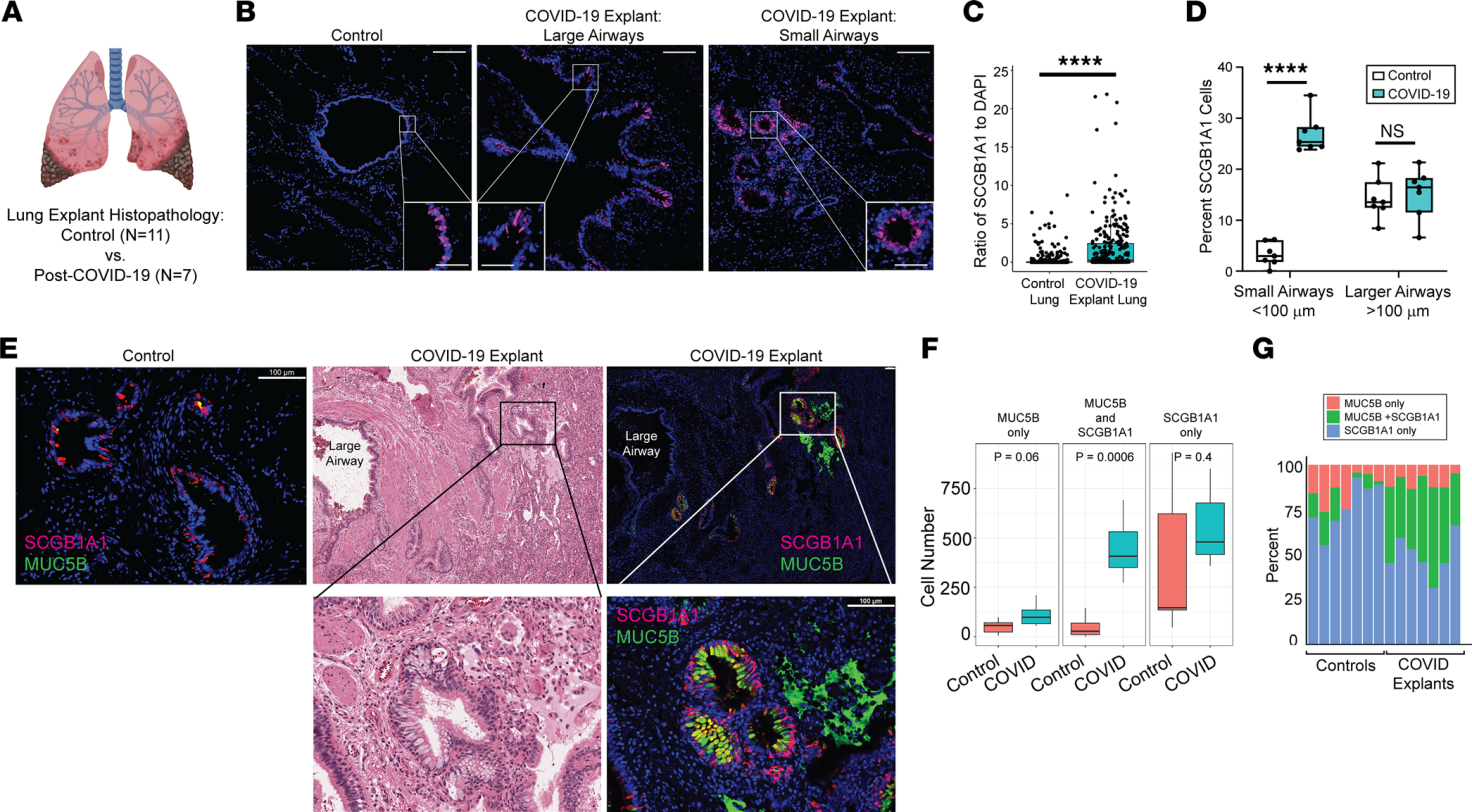
Increased SCGB1A1 immunofluorescence with MUC5B in small airways of the lung after severe COVID-19. (**A**) Schematic. (**B**) Immunofluorescence pattern of SCGB1A1 in large and small airways in control and COVID-19 explants (scale bars: 200 μm). Insets show staining at higher magnification (scale bars: 50 μm). (**C**) Automated quantification of SCGB1A1 immunofluorescence in control (*n* = 11) and COVID-19 explanted lungs (*n* = 7). Each point represents the average SCGB1A1^+^ areas divided by number of DAPI^+^ cells of 42 photomicrographs for each case at ×20 magnification. ****P < 2.2 × 10^–16^. (**D**) Manual counting of SCGB1A1^+^ airway epithelial cells in airways measuring less than or greater than 100 μm in diameter in 4 slides from all *n* = 7 COVID-19 explants and *n* = 7 sex-matched controls. ****P < 1 × 10^–6^ for small (<100 μm) airways. *P* = 0.67 for large airways. (**E**) Representative coimmunofluorescent staining of SCGB1A1 and MUC5B in control and COVID-19 explant tissue (scale bars: 100 μm). H&E staining of an adjacent cut from the COVID-19 explant is shown for reference. Higher-power magnification images of the H&E-stained slide and the immunofluorescence image from the areas denoted by the boxes are shown below. MUC5B signals (green) are seen not only in cells but also within the adjacent extracellular space. (**F**) Quantification of the absolute number of MUC5B^+^ cells, SCGB1A1^+^ cells, and MUC5B^+^SCGB1A1^+^ double-positive cells in COVID-19 explants (*n* = 7) and control lung tissue (*n* = 7). (**G**) Bar graph demonstrating the percentage of airway cells with the indicated staining pattern found in COVID-19 explants (*n* = 7) and control lung tissue (*n* = 7). *P* values are from Mann-Whitney tests.

We compared lung explants from 7 individuals who underwent lung transplantation 2–29 months after COVID-19 (Supplemental Table 10) to 11 normal control samples (lung resected adjacent to a pulmonary nodule) (Figure 6A). There was no difference between SCGB1A1/CC16 immunofluorescent staining of epithelial cells lining large (>100 μm) airways, but the post–COVID-19 lung explants showed dramatically increased expression in low cuboidal epithelium lining small (<100 μm) airways (Figure 6, B–D). Compared with control samples, COVID-19 explant samples (Figure 6E) had a greater than 3-fold increase in CC16 and MUC5B coexpression (0%–14% vs. 18%–26% cells, respectively; *P* = 0.0006) (Figure 6, F and G). These double-positive cells were typically located in small airways in areas of peribronchial metaplasia, with MUC5B staining the cell apices and extracellular spaces, and CC16 staining the basolateral portion of the cells. Photomicrographs of immunofluorescence antibody controls are shown in Supplemental Figure 8.

## Discussion

In 3 racial and ethnically diverse cohorts of adults hospitalized during the wild-type and Delta waves of the COVID-19 pandemic, we found consistent cross-sectional and longitudinal associations of higher circulating CC16 associated with fibrosis-like abnormalities on thoracic CT for up to 3 years after hospitalization. CC16 appears to have a dose-response association with larger airway-to-lung ratio, suggesting that its secretion may be proportional to cellular changes from SARS-CoV-2–mediated airway injury and repair. Our transcriptomic and immunofluorescence analyses confirm increased numbers of small airway epithelial cells expressing SCGB1A1/CC16 and coexpressing MUC5B in patients with post–COVID-19 fibrotic lung abnormalities. Collectively, our findings suggest that CC16 is a postacute circulating biomarker of COVID-19–related epithelial injury that represents pathologic remodeling of small airway epithelium associated with persistent fibrosis.

Human proteome maps show that CC16 expression occurs primarily in the lung epithelium. Experimental and clinical studies demonstrate that CC16 is a pleiotropic negative regulator of inflammation. While the mechanisms by which CC16 decreases lung inflammation are not completely understood, CC16 attenuates acute inflammation via limiting neutrophil chemotaxis and limits chronic inflammation via inhibition of osteopontin, which stimulates epithelial cell production of T helper cell 2 cytokines (27, 28). Consistent with these experimental studies, higher levels of circulating CC16 are associated with less severe chronic obstructive pulmonary disease (COPD) and a slower decline in forced expiratory volume in 1 second (FEV1) (29). Increased CC16 is observed in non-COVID ARDS and in severe COVID-19 (30, 31), presumably reflecting lung epithelial cell injury and alveolar–blood barrier leakage. Higher circulating CC16 levels are also found in multiple restrictive interstitial lung diseases (27, 32, 33), where it may reflect a response to ongoing lung injury and repair. For example, in a cohort study of IPF patients (32), mean (±SD) serum CC16 level was 31,200 ± 10,800 ng/mL. In our discovery cohort, mean (±SD) serum CC16 at 4 months and 15 months was higher than in these IPF patients (44,800 ± 53,700 and 52,800 ± 69,100 ng/mL, respectively), and was observed to be similar to these IPF patients at 3 years (31,900 ± 29,700 pg/mL). Additionally, our observation of an inverse correlation between CC16 and DLCO suggests that the increase in SCGB1A1 cells is linked to functional limitations, including decreased diffusion at the alveolar capillary interface. Our investigation is consistent with these previous studies insofar as CC16 levels are highest at hospital discharge, closest in time to COVID-19 ALI. Its gradual decline but persistent relative elevation in those with fibrosis-like abnormalities on thoracic CT is consistent with it being a measure of the degree of lung epithelial damage and subsequent repair of the distal small airways associated with persistent fibrosis.

The 2 SCGB1A1^+^ epithelial cell clusters identified by unsupervised clustering have been characterized as airway progenitor cells (34, 35). The SCBG1A1^+^SCGB3A2^+^SFTPB^+^ cell cluster, termed a RAS cell, can self-renew and generate AT2 cells (25, 26). Similarly, SCGB1A1^+^ secretory cells can self-renew and contribute to airway epithelial repair after injury (36). The subset of SCGB1A1 cells that coexpresses MUC5B are normally found by immunohistochemistry to occupy the mid-portion of the human airway tree (25). But in this study, we found approximately 3-fold more SCGB1A1^+^MUC5B^+^ cells anomalously located in the small (<100 μm) respiratory bronchioles in post–COVID-19 fibrosis. Interestingly, a recent scRNA-seq analysis also revealed enriched populations of secretory cell clusters with transcriptional signatures that include *SCGB1A1*, *SCGB3A2*, and *MUC5B* in IPF (37), suggesting that dual activation of alveolar and secretory epithelial repair programs are characteristic not only of IPF but also post-COVID fibrosis.

It is biologically plausible that persistence of SARS-CoV-2 contributes to the localization of fibrotic remodeling within the respiratory bronchioles. Single-nucleus transcriptomic analyses of the human lung demonstrate high expression of the SARS-CoV-2 ACE2 receptors and TMPRSS2 entry factor across multiple epithelial cell populations, including AT1 and AT2 cells, as well as club, goblet, and ciliated cells (38). Notably, expression of these is further upregulated following viral infection, suggesting a positive feedback mechanism that may enhance viral tropism and persistence within distal airway epithelium. Consistent with this hypothesis, prolonged SARS-CoV-2 replication has been reported to persist for up to 4 weeks after symptom onset (39). Future studies are needed to determine whether persistent viral replication within the small airways drives aberrant repair responses and fibrotic remodeling in post–COVID-19 lung disease.

The rs3570590 MUC5B promoter variant confers an inherited risk factor for IPF and other types of pulmonary fibrosis by increasing MUC5B expression (40–44). This study found large numbers of SCGB1A1^+^MUC5B^+^ secretory epithelial cells that persist years after the initial SARS-CoV-2 infection and aberrantly reside in elongated respiratory bronchioles, thus activating a profibrotic program in the most distal aspects of the human airway tree. Although the COVID-19 survivors with fibrosis did not carry this genomic risk allele, we found evidence for increased numbers of SCGB1A1^+^MUC5B^+^ cells by immunofluorescence, suggesting that viral infection may lead to acquired MUC5B overexpression. Thus, there may be convergence of genetic and virally induced MUC5B overexpression that both lead to an increased risk of fibrosis. As this variant is associated with decreased hospitalization and improved survival after COVID-19 (2, 45), it may have been subject to natural selective pressure from preceding waves of deadly respiratory viral infections leading to its enrichment in European populations.

The identification and scoring of fibrosis-like residual abnormalities by thoracic radiologists, particularly traction bronchiectasis, can be difficult and subjective. Therefore, we took what we believe to be a novel approach of validating a dose-response association of CC16 with fibrosis-like abnormalities using airway-to-lung ratios calculated from supervised machine-learning software. Previously, lower airway-to-lung ratio, as an index of size dysanaptic lung growth, revealed an association with COPD risk among community-dwelling older adults (46). Our finding that higher circulating CC16 levels are linearly associated with greater airway-to-lung ratio suggests that the level of circulating CC16 may reflect the extent of postinfection remodeling of airway architecture, as evidenced by shifts in the proportions of epithelial cells observed in our transcriptomic analyses and the robust expression of SCGB1A1 in the small airways of COVID-19 survivors in our histopathologic analyses. These lung architectural changes may manifest as traction bronchiectasis, bronchiolectasis, and an increased airway-to-lung ratio.

While CC16 demonstrated the most consistent and robust associations with fibrosis-like patterns over 3 years, we also observed independent associations of higher levels of COMP at hospital discharge with fibrotic changes at 15 months and 3 years, and higher levels of GDF-15 at hospital discharge with fibrotic changes at 3 years. Increased COMP expression in the early postacute phase of illness at hospital discharge may contribute to fibrotic remodeling by promoting extracellular matrix deposition and sustained fibroblast activation. GDF-15 expression is increased in ALI (47), and persistent elevations in circulating GDF-15 at hospital discharge could represent ongoing epithelial injury that may drive airway remodeling and fibrosis. Future studies could consider investigating these biomarkers at hospital discharge and in the weeks thereafter as early indicators of post–COVID-19 lung fibrosis.

Our study design has limitations. The sample sizes of our study cohorts are modest, ranging in size from 37 to 150 participants. Accordingly, we lack statistical power to test longitudinal mixed-effects models with patient-specific intercepts and to detect significant associations in the smallest validation cohort. Each cohort had differences in demographics, the prevalence of invasive mechanical ventilation use, and methods to assess for persistent lung abnormalities on chest CT, which likely account for some variation in the observed prevalence of reticulations and traction bronchiectasis across cohorts. Despite these differences, our consistent associations of CC16 with fibrosis-like abnormalities suggest that our results are robust to variation in case-mix and measurement. The observed effect sizes in the discovery cohort may overestimate what would be observed in a larger population of COVID-19 survivors since we oversampled invasive mechanical ventilation survivors, who likely had the highest CC16 levels acutely and who are at the greatest risk for post-COVID residual fibrotic lung abnormalities (7, 13, 24). Conversely, conditioning on survival in a cohort enriched with mechanical ventilation survivors could attenuate associations for biomarkers reflecting acute severity rather than repair pathways. Thus, the robust CC16 associations observed in the discovery cohort arguably strengthens the inference that CC16 captures fibrosis-specific biology orthogonal to acute severity. Potential selection bias from oversampling of mechanical ventilation survivors in the discovery cohort could have also masked associations with other biomarkers observable in more diverse populations.

Our study has other limitations. We did not test associations of pulmonary epithelial cell proteins, such as metalloproteinase-7 (MMP-7), Krebs von den Lungen-6 (KL-6), surfactant protein-A (SPA), and surfactant protein-D (SP-D) that have been linked to IPF, and some of which have recently been found to be associated with reduced lung diffusion capacity in COVID-19 survivors (48). We could not confirm whether the “control” transplanted lung or the lung resections had prior SARS CoV-2 infection that resolved without radiographic or histopathologic abnormalities. While the inclusion of lung transplant recipients as controls for the single-cell analysis may have had transcriptional changes related to their immunosuppression medications, the findings relevant to CC16/SCGB1A1 were also replicated in the immunofluorescence analysis of explant tissue and control lung resections adjacent to nodules in non-immunosuppressed individuals. Thus, the persistent transcriptomic and immunohistochemical changes that we identified in COVID-19 survivors with fibrosis-like abnormalities may reflect other sequelae of SARS-CoV-2 infection itself. The numbers of participants and tissue available for the scRNA-seq and immunohistochemical analyses, although age- and sex-matched, are limited and may be subject to sampling bias. The prevalence and extent of fibrotic changes 1 or more years after non-COVID ARDS in the low tidal volume ventilation era is less that observed in COVID-19 ALI/ARDS (49, 50), but is still occasionally observed. Whether our finding of elevated CC16 and dysregulated airway epithelial progenitor cell remodeling is specific to COVID-19 or a generic response in all ALI/ARDS survivors with fibrotic changes remains unknown. Cohort enrollment occurred primarily during the wild-type wave of the SARS-CoV-2 pandemic, with some McGill participants sampled from the early Delta strain wave. Whether our findings remain consistent with vaccination or subsequent SARS-CoV-2 variant infection remains unknown. Future studies in larger population-based cohorts are needed to investigate the potential clinical utility of using CC16 levels to prognostically enrich and facilitate screening of COVID-19 survivors for fibrotic lung abnormalities.

In conclusion, CC16 remains consistently elevated and associated with thoracic CT fibrosis-like abnormalities for 3 years after hospitalization among adult survivors of moderate-to-critical COVID-19. Elevated circulating CC16 appears to reflect underlying deranged pulmonary epithelial progenitor proliferation and anomalous CC16/MUC5B-related fibrotic signaling in distal human airways. These findings suggest that CC16 should be investigated further as a blood biomarker that may be leveraged to facilitate screening of COVID-19 survivors for persistent radiographic abnormalities and their functional consequences.

## Methods

### Sex as a biological variable.

Sex was considered as a biological variable. Both men and women who were hospitalized for acute COVID-19 and survived were invited to provide consent to participate in the cohort studies.

### Study design and participants.

The Columbia-based discovery cohort has been described in detail previously (7, 13, 24). We prospectively enrolled ambulatory community-dwelling adults from New York City 21 years of age or older hospitalized at the Columbia tertiary-care Milstein Hospital and community-based Allen Hospital with laboratory-confirmed SARS-CoV-2 infection and severe or critical COVID-19 between March 1 and May 15, 2020. We excluded those with a history of interstitial lung disease or lung transplantation. We weighted sampling to include approximately 50% who required invasive mechanical ventilation during their COVID-19 hospitalization. We enrolled a total of 150 participants. Seventy-six participants were initially enrolled at 4 months after hospital discharge and invited to participate in 15-month and 3-year follow-up. We prospectively enrolled 47 and 27 additional participants with the same inclusion criteria and 50% sample weighting for requiring mechanical ventilation to increase and then maintain the sample size at approximately 100 participants at 15-month and 3-year follow-up. In previous analyses, we found that demographic, genomic, comorbidity, and acute COVID hospitalization characteristics did not differ between those who did and did not follow-up across study visits, suggesting that cohort attrition was not affected by differential loss to follow-up (13, 24).

For all study cohorts, fibrosis-like abnormalities were defined as reticulations, traction bronchiectasis, or honeycombing.

We compared single-cell analyses of transbronchial lung biopsies of fibrosis-like abnormalities in study participants obtained between 3 and 4 years after acute COVID-19 to transbronchial biopsies from 4 asymptomatic lung transplant recipients done as part of routine surveillance during the first year after lung transplantation. Since there was no evidence of infection or rejection and normal appearing lung architecture in the lung transplant biopsies, these samples were used as healthy lung tissue controls. We conducted immunofluorescence studies of explanted lungs from 7 adults who underwent lung transplantation for COVID-19 pulmonary fibrosis, and non-diseased lung tissue of 11 adults who underwent surgical resection of pulmonary nodules. A thoracic pathologist reviewed all transbronchial biopsies and explant lung tissues.

The UBC validation cohort has been described in detail previously (51). Adults with COVID-19 hospitalized in Vancouver, Canada between March and May, 2020 with laboratory-confirmed SARS-CoV-2 infection were prospectively enrolled with thoracic CT scans and plasma biobanking at 3 months. Patients with preexisting interstitial lung disease were excluded.

The McGill validation cohort was sampled as a nested case-control study cohort from the Biobanque Québécoise de la COVID-19 cohort (https://www.bqc19.ca/), since only some participants received posthospitalization thoracic scans for clinical care (52). We sampled adults hospitalized with laboratory confirmed SARS-CoV-2 infection and COVID-19 from the Jewish General Hospital (JGH) in Montréal, Québec, Canada, excluding those with fibrosis-like abnormalities on thoracic CT scans within 5 years prior to the SARS-CoV-2 pandemic, interstitial lung disease, lung transplantation, and end-stage renal failure. Cases were defined as patients who were hospitalized with COVID-19 between March, 2020 and November, 2021 and had fibrosis-like abnormalities noted on clinical reports of thoracic CT scans assessed either within 1 week of hospital discharge, or approximately 4 months or 15 months after hospital discharge. Controls were sampled with the same inclusion criteria in a 2:1 ratio to cases and defined as patients with no mention of fibrosis-like abnormalities on CT scans obtained at similar follow-up intervals. After finding a higher prevalence of honeycombing than in the other cohorts, we re-reviewed the 4 cases with honeycombing on post–COVID-19 thoracic CT. We conducted a post hoc sensitivity analysis, excluding 3 cases who were ultimately found to have possible honeycombing and lung distortion on thoracic CT done more than 5 years prior to the SARS-CoV-2 pandemic.

### Thoracic CT analyses.

For the Columbia discovery cohort, we obtained non-contrast high resolution CT scans at maximal inspiration at 4 months, 15 months, and 3 years after hospitalization for COVID-19. Two chest radiologists evaluated each thoracic CT, as previously described (7, 13, 24). We examined 15-month thoracic CT scans to assess airway-to-lung ratio for patho-radiographic analyses. Airway lumen diameters were assessed at 19 standard anatomic locations (trachea to subsegments) and total lung volumes were segmented and measured from inspiratory thoracic CT images using Apollo software from VIDA Diagnostics by trained technologists unaware of other participant information. We calculated airway-lung ratios using previously established methods (46). Trained technologists were unaware of participant other information. At least 1 subsegmental lumen (see Figure 4A) either did not exist or was not visualized by the Apollo software among 22 of 65 15-month participants with CC16 measured at hospital discharge, 14 of 54 15-month participants with CC16 measured at 4 months, and 32 of 96 15-month participants with CC16 measured at 15 months. In our primary analysis (Figure 4), we report the geometric mean allowing for missing subsegmental values. We conducted 2 sensitivity analyses to ensure that the observed results are robust to small airway detection bias: (a) we conducted a complete-case analysis not allowing for missing subsegmental values and (b) we assessed the geometric mean from trachea to segments, excluding subsegments entirely (Figure 4E).

For the UBC validation cohort, we obtained high-resolution thoracic CTs. Two cardiothoracic radiologists independently evaluated radiographic abnormalities by separating each lung into 3 zones and estimating the percentage of lung affected by either ground glass or reticulation. The presence versus absence of traction bronchiectasis and honeycombing was also noted in each lung zone. Further details have been described previously (51).

In the McGill validation cohort, thoracic CT scans were obtained for clinical care. Fibrosis-like abnormalities were defined as a mention of reticulations, traction bronchiectasis, or honeycombing in the clinical report.

### Biobanking and biomarker analyses.

For the Columbia and UBC cohorts, we prospectively obtained peripheral blood samples at follow-up. Prospectively collected blood samples were centrifuged immediately, and supernatant was aliquoted and cryopreserved at –80°C. Hospital discharge samples for the Columbia cohort were obtained from remaining blood samples used for clinical care within 7 days of hospital discharge (median –2, IQR [–3.5 to –1] days). The UBC cohort had no hospital discharge samples. Among McGill participants, we assessed the plasma sample closest in time to the date of hospital discharge, with a limit of ±10 days (observed median –2, IQR [–1 to –7]). The McGill cohort had no samples at posthospitalization follow-up. Serum and plasma levels of biomarkers were assessed in the discovery and validation cohorts, respectively. Therefore, biomarker levels cannot be compared between discovery and validation cohorts.

All biomarker assessments were performed in duplicate at the Columbia University Irving Institute for Clinical and Translational Research using Miliplex multiplex assays with the Luminex xMAP bead-based multiplex assay platform (MiliporeSigma). We assessed biomarkers from the soluble cytokine receptor panel, human cardiovascular panel 2, human cytokine/chemokine/growth factor panel A, human angiogenesis/growth factor 1, and human fibrosis panel (which includes CC16, also known as uteroglobin). For CC16, the median [IQR] coefficients of variation for CC16 were 3.3% [1.8%–6.6%], 4.5% [2.4%–9.7%], 3.3% [1.8%–6.6%], and 4.6% [2.2%–10%]) at hospital discharge, 4-month, 15-month, and 3-year Columbia cohort samples, and 5.2% [2.4%–9.2%] and 5.4% [3.1%–8.4%] for the UBC and McGill cohort samples, respectively.

### FACS of lung transbronchial biopsy samples.

We employed FACS to separate and quantitate populations of lung cells prior to scRNA-seq. A cell suspension was prepared from transbronchial lung biopsies by digestion in 1.4 mL DMEM containing 0.3 mg/μL Liberase (Sigma-Aldrich), 70 μg/μL elastase (Worthington), and 3 U/mL Dispase II (Sigma-Aldrich) at 37°C for 45 minutes, followed by the addition of 10 μL DNase (2.7 U/μL; Qiagen), 1 μL 1 M DTT, and 2 μL 0.5M EDTA, and incubation for an additional 15 minutes. The cell suspension was strained through a 100-μm filter, centrifuged at 300*g* for 10 minutes, and resuspended in approximately 200 μL residual buffer plus 10 μL DNase. Red blood cells were lysed by adding 1 mL ACK buffer (Gibco) and incubating on ice for 2 minutes. Following the addition of 10 mL PBS with 1% BSA, centrifugation and resuspension in approximately 200 μL residual buffer plus 10 μL DNase, the cells were blocked with FcR Blocking Reagent (Miltenyi Biotec) and stained 1:100 with CD326/EpCAM–Alexa Fluor 488 (clone MH99, Invitrogen/eBioscience, catalog number 53-8326-42), CD31–APC (clone WM59, BioLegend, catalog number 303115), and CD235a/Glycophorin A–PE (clone HI264, BioLegend, catalog number 349105) for 1 hour at 4°C, strained through a 100-μm filter, and flow sorted on a BD Influx FACS instrument. We positively sorted for complex, singlet cells and negatively sorted for DAPI and PE, which removed the dead cells and any remaining red blood cells. The FACS-isolated cells were collected as 3 populations: CD326/EpCAM-positive (epithelial), CD31/CD45-positive (immune and endothelial), and those negative for either AF488 or APC fluorophore signals (enriched for fibroblasts). Using the cell count numbers obtained by FACS, each of the 3 populations of cells were mixed 1:1:1, using the population with the fewest number of cells as the limiting factor. Following centrifugation and resuspension in PBS with 0.04% BSA, cell viability was measured using trypan dye exclusion, and 10,000 cells were loaded on a 10X Genomics flow cell for encapsulation in individual droplets for lysis, reverse transcription, and barcoding of cDNA, followed by sequencing on an Illumina NovaSeq 6000.

### scRNA-seq analysis.

Postprocessing analysis was performed using Seurat v4.4.0 (53). Cells were filtered using the *Subset* function to remove those with >10% mitochondrial genes and <200 or >7500 genes expressed and <4000 or >40000 transcripts identified. Sample-level datasets were integrated using reciprocal PCA. The *NormalizeData* function was used to log normalize data and the *ScaleData* function was used to regress out the number of UMIs and percentage of mitochondrial genes. Principal component analysis was performed using *RunPCA* and Louvain clustering was performed using the *FindNeighbors* and *FindClusters* functions. UMAP embeddings were calculated using *RunUMAP* for visualization. Correlation plots of the proportion of cell clusters in control and COVID-19 samples were graphed using the R package *CorrPlot* (https://cran.r-project.org/package=corrplot).

To interpret cellular compositions of each sample, we grouped all annotated cell types by FACS groups corresponding to epithelial cells (EpCAM^+^), endothelial/immune cells (CD31^+^CD45^+^), and stromal or other cells (EpCAM^–^CD31^–^CD45^–^). We visualized the transcriptomic expression of *EPCAM*, *PTPRC* (CD45), and *PECAM1* (CD31) in each cell type to confirm correct membership. We employed a combination of an unsupervised and supervised approach to determine cell type annotation. We identified top genes that were unique to each cell cluster using *FindMarkers* using ROC analysis.

### Immunofluorescent staining and image analysis of lung explant samples.

Formalin-fixed paraffin-embedded (FFPE) lung slides were dewaxed using xylene (3 times, 5 minutes each), rehydrated using gradients of ethanol (100%, 95%, 70%, and 50% ethanol; 2 times, 10 minutes each), and washed with deionized water (2 times, 5 minutes each). For quantification of SCGB1A1 immunofluorescence, antigen retrieval was performed by immersing the slide in 10 mM sodium citrate buffer (pH 6.0) at 95°C and then cooling to room temperature for 30 minutes. The slides were washed in distilled water for 5 minutes, permeabilized by 0.1% Triton X-100 in Tris-buffered saline (TBS) for 15 minutes, and washed with TBS (2 times, 5 minutes each). The slides were blocked using 5% donkey serum in TBS with 0.1% saponin (TBSS) for 60 minutes at room temperature and then incubated overnight at 4°C with primary antibody (rabbit anti-SCGB1A1; ProteinTech catalog number 10490-1-AP, 1:200). The slides were washed 3 times (5 minutes each) with TBSS and incubated with secondary antibody at 1:500 for 60 minutes at room temperature. The slides were washed 2 times (5 minutes each) with TBSS and mounted using DAPI Fluoromount-G (Southern Biotech, 0100-20). For quantification of both SCGB1A1 and MUC5B, the above protocol was followed with the following changes: antigen retrieval was performed using 10 mM sodium citrate buffer (pH 9.0) with 0.05% Tween 20, slides were blocked using 5% goat serum in TBSS as above and then incubated overnight at 4°C with primary antibody (mouse anti-SCGB1A1, Santa Cruz Biotechnology, catalog number SC-365992, 1:100; rabbit anti-MUC5B, Millipore Sigma, catalog number HPA 008246, 1:100; or both). Fluorescence images were taken using a Leica widefield fluorescence microscope.

Automated quantification of immunofluorescence imaging was performed using ImageJ (NIH). Monochrome images were thresholded to ensure removal of background autofluorescence. The percentage of SCGB1A1-stained cells was quantified using the *Analyze Particles* command by setting the size threshold to 1 μm^2^ and using all other default settings. Counting DAPI^+^ cells was performed in a similar manner. For each slide, the ratio of SCGB1A1^+^ particle area to DAPI^+^ particle number was calculated for each of 42 images (×20 magnification) from each control and COVID-19 lung sample. One-tenth of the images with SCGB1A1^+^ cells from each sample were randomly selected and analyzed by manually counting the number of SCGB1A1^+^ airway epithelial cells in airways measuring >100 μm or <100 μm in diameter. Manual counting of the number of SCGB1A1^+^, MUC5B^+^, and SCGB1A1^+^MUC5B^+^ airway epithelial cells in COVID-19 explants and sex-matched controls (*n* = 7 each) was performed using 5 × 5 tile scans.

### Genotyping.

DNA was isolated from each Columbia participant at follow-up and DNA was isolated from blood leukocytes using the Gentra Puregene Blood Kit (Qiagen). Genotyping for the *MUC5B* rs35705950 risk allele was obtained by Sanger sequencing.

### Statistics.

We examined unadjusted associations of biomarkers and clinical characteristics with Student’s *t*, Mann-Whitney, χ^2^, Fisher’s exact, ANOVA, and Kruskal-Wallis tests. We created separate GAMs with locally weighted smoothing to assess for nonlinear adjusted associations between biomarkers and the predicted risk of fibrosis-like abnormalities. We natural log–transformed right-skewed biomarker data. We used covariate balancing propensity scores (CBPS) to adjust for covariables to avoid overparameterized models at our study sample size, as we have done previously (7, 54). Covariates included in CBPS were age, sex, race/ethnicity, body mass index (BMI), smoking history, COPD, asthma, estimated glomerular filtration rate, use of corticosteroids during COVID-19 hospitalization, IL-6 receptor inhibitor therapy during COVID-19 hospitalization, ventilator days during COVID-19 hospitalization, and days between SARS-CoV-2 infection and thoracic CT scan. We estimated adjusted ORs per natural log(fold change) in CC16 or by tertiles of CC16 using logistic regression. In analyses described in Figure 1, we adjusted for a false discovery rate (FDR) of 0.05 based on biomarker class and category of association using the Benjamini-Hochberg method (55). To ensure that our results were robust to potential model overfitting, we examined all GAM plots and conducted sensitivity analyses excluding visible outliers >2 SDs that persisted after natural log transformation. Analyses were performed using Stata v16 (StataCorp) and R studio (v2024.04.2+764).

### Study approval.

All studies at Columbia University were approved by their Institutional Review Board (IRB) (AAAT5605, AAAT0009, and AAAS0753). The cohort study at UBC was approved by their IRB (no. H20-01239). Columbia and UBC participants or a legally authorized representative signed written informed consent. At McGill, consent was obtained based on the BQC19’s standard operating procedures, which were approved by their IRB (52).

### Data availability.

Values for CC16 and presence versus absence fibrotic abnormalities for each participant in each cohort are available in the Supporting Data Values file. scRNA-seq data have been deposited in the NCBI Gene Expression Omnibus database (GEO GSE208611). Other data are not publicly available due to them containing personally identifiable information that could compromise research participant privacy and consent. Data sharing will require an IRB-approved data sharing agreement. Contact Matthew R. Baldwin (mrb45@cumc.columbia.edu) for Columbia University discovery cohort data inquires, Christopher Carlsten (christopher.carlsten@ubc.ca) for University of British Columbia validation cohort data inquires, and Tomoko Nakanishi (tomoko.nakanishi@mail.mcgill.ca) for McGill data from Biobanque Québécoise de la COVID-19 cohort data inquires.

## Author contributions

The manuscript was initially drafted by MRB, with support from CKG. MRB, CKG, CC, CJR, JBR, and TN made substantial contributions to the conception and design of the work. AEJ, SOM, CFM, CKG, ACYY, PJ, AWW, ASS, and TN have accessed and verified data of the respective cohorts. ASS reviewed all transbronchial biopsies and explant lung tissues. RN had oversight of the plasma and serum biomarker assessments. All authors contributed to data interpretation, critical review and revision of the manuscript, and final approval of the version to be published. All authors are responsible for the decision to submit the manuscript and are accountable for all aspects of the work in ensuring that questions related to the accuracy or integrity of any part of the work are appropriately investigated and resolved.

## Conflict of interest

The authors have declared that no conflict of interest exists.

## Funding support

This work is the result of NIH funding, in part, and is subject to the NIH Public Access Policy. Through acceptance of this federal funding, the NIH has been given a right to make the work publicly available in PubMed Central.

- US Department of Defense (W81XWH2110217 to MRB and W81XWH2110216 to CKG).
- NIH (UL1TR001873 to MRB, R01HL103676 and S10OD032447 to CKG).
- Japan Society for the Promotion of Science for Young Scientists (22KJ1190 and 22J30004 to TN).
- Grant-in-Aid for Scientific Research (JSPS KAKENHI grant number 23H02917 to TN).

## Supplementary Material

Supplemental data

ICMJE disclosure forms

Supporting data values

## Figures and Tables

**Table 1:**
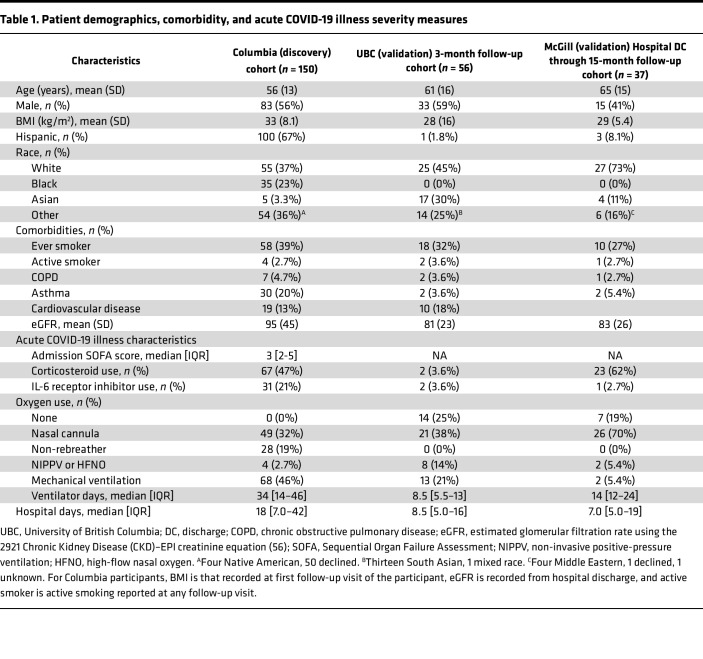
Patient demographics, comorbidity, and acute COVID-19 illness severity measures

## Version 1 07/08/2026

Electronic publication
