# Supplementary material for: Pulmonary fibrosis after COVID-19 is characterized by airway abnormalities and elevated club cell secretory protein-16
Source: JCI Insight. 2026 Jul 8;11(13):e199983. doi: 10.1172/jci.insight.199983 (PMC13460843; doi:10.1172/jci.insight.199983)
Supplement: Supplementary file 1 [file jciinsight-11-199983-s252.pdf]

Online Data Supplement

Pulmonary fibrosis after COVID-19 is characterized by airway abnormalities with persistently elevated CC16

Matthew R. Baldwin, MD, MS; Ansley E. Jones, MHS; David Zhang, MD; Chandan Gurung, PhD; Zain Khan, BS; Anjali Saqi, MD; Xuehan Yang, MS; Ying Wei, PhD; Renu Nandakumar, PhD; Scarlett O. Murphy, MD; Claire F. McGroder, MD, MS; Faisal Shaikh, MD; Selim Arcasoy, MD; Luke Benvenuto, MD; Harpreet Grewal, MD; Benjamin M. Smith, MD; Eric A. Hoffman, PhD; Agnes CY Yuen, BSc; Parteek Johal, BSc; Chrisopher Carlsten, MD, MPH; Christopher J Ryerson, MD; J. Brent Richards, MD, MSc; Alyson W Wong, MD; Tomoko Nakanishi, MD, PhD; Aditi S Shah, MD Christine K. Garcia, MD, PhD

TABLE OF CONTENTS (in order of citation in the main manuscript)

Materials

Supplement Figure 1. Columbia (discovery) cohort follow-up visit participation.

Supplement Table 1. Demographic and clinical characteristics stratified by follow-up visit in the Columbia (discovery) cohort.

Supplement Table 2. Fibrotic-like abnormalities on Thoracic CT scans in the Columbia (discovery), University of British Columbia (validation), and McGill (validation) cohorts.

Supplement Table 3. Comparison of demographic and clinical characteristics between those with and without fibrotic-like abnormalities in the McGill (validation) nested case-control cohort.

Supplement Figure 2. Longitudinal plots of individual CC16 levels among Columbia (discovery cohort) (A) 4-month participants with subsequent follow-up visits and (B) 15-month participants with subsequemt follow-up visits.

Supplement Table 4. Effect estimates of associations between CC16 and fibrotic-like lung abnormalities on thoracic CT scan in the Columbia (discovery) cohort.

Supplement Table 5. Cross-sectional spearman correlations of CC16 and percent predicted diffusion capacity of lung for carbon monoxide (DLCO) at follow-up in the Columbia (discovery) and University of British Columbia (validation) cohorts.

Supplement Table 6. Unadjusted and adjusted cross-sectional associations of telomere length and CC16 levels at 4 months, 15 months, and 3 years in the Columbia (discovery cohort).

Supplement Table 7. Effect estimates of associations between CC16 and fibrotic-like lung abnormalities on thoracic CT scan in the University of British Columbia and McGill (validation) cohorts.

Supplement Figure 3. Circulating CC16 (pg/ml) levels in those with and without fibrotic-like abnormalities in the McGill (validation) nested case-control cohort, stratified by follow-up time period of thoracic CT scan: (A) hospital discharge, (B) 4 months; (C) 15 months.

Supplement Table 8. Generalized linear model adjusted associations of CC16 with airway-to-lung ratio 15 months in Columbia (discovery) cohort.

Supplement Figure 4. Circulating CC16 (pg/ml) levels in those with and without fibrotic-like abnormalities in the McGill (validation) nested case-control cohort sensitivity analysis excluding n=3 case patients with honeycombing in the McGill cohort (A), and stratified by follow-up time period of thoracic CT scan: (B) hospital discharge, (C) 4 months; (D) 15 months. Sensitivity analysis. Generalized additive model (GAM) with LOESS smoothers with adjustment for age,

sex, race/ethnicity, BMI, COPD, pack-year history of smoking, estimated glomerular filtration rate, ventilator days, and days since initial SARS-CoV-2 infection using covariate balanced propensity scores.

Supplement Figure 5. Sensitivity analyses of generalized additive model adjusted associations between CC16 and airway to lung ratio. (A-C) Complete case analyses among those with all subsegmental airways visualized. (D-F) Analyses of all subjects excluded sub-segmental measurements.

Supplement Figure 6. Histology of lung samples included as controls for the single cell RNA sequencing analysis.

Supplement Table 9. Demographics and clinical characteristics of COVID-19 survivors with fibrosis on thoracic imaging at 3-year follow-up and lung transplant receipts who underwent transbronchial biopsies

Supplement Figure 7. Single cell RNA sequencing quality control, gene expression data, and differences in cell numbers of immune, endothelial, and non-epithelial, non-endothelial, and non-immune cells

Supplement Table 10. Demographics of patients who underwent lung transplantation for COVID-19 pulmonary fibrosis and controls who underwent surgical resection of pulmonary nodules.

Supplement Figure 8. Immunofluorescence antibody controls.

Supplement References

Materials

Antibodies include Rabbit anti-SCGB1A1 (Cat#10490-1-AP, Proteintech); Mouse anti-SCGB1A1 (Cat# sc-365992, Santa Cruz), Rabbit anti-MUC5B (Cat# HPA008246; Sigma-Aldrich), Donkey anti-rabbit IgG (H+L) Alexa Fluor Plus 647 (Cat#A32795, Invitrogen), Goat anti-mouse IgG (H+L) Alexa Fluor 488 (Cat#A11001, Invitrogen).

Supplement Figure 1. Columbia participant follow-up visit participation.

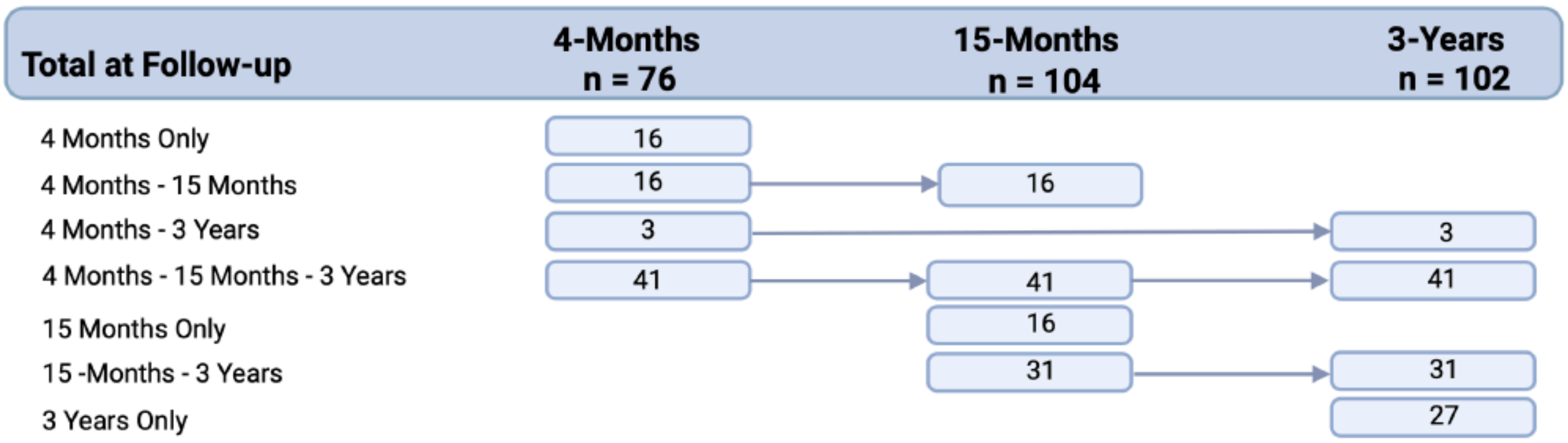

Supplement Table 1. Demographic and clinical characteristics stratified by follow-up visit in the Columbia (discovery) cohort.

Characteristics	Columbia 4-month follow-up (n=76)	Columbia 15-month follow-up (n=104)	Columbia 3-year follow-up (n = 102)
Age, years, mean (SD)	54 (14)	54 (11)	56 (12)
Male	45 (61%)	61 (59%)	55 (54%)
Body Mass Index (kg/m^2), mean (SD)	32 (6.9)	32 (6.8)	32 (6.2)
Hispanic, n (%)	43 (57%)	62 (60%)	69 (68%)
Race, n (%)			
White	30 (39%)	30 (29%)	40 (40%)
Black	22 (30%)	28 (27%)	25 (25%)
Asian	1 (1%)	4 (4%)	4 (4%)
Other	23 (30%)	42 (40%)	33 (32%)
**Comorbidities**			
Ever smoker*	31 (41%)	40 (38%)	39 (38%)
Active smoker	2 (3%)	3 (3%)	2 (2%)
COPD	4 (5%)	4 (4%)	2 (2%0
Asthma	18 (27%)	24 (23%)	22 (22%)
Cardiovascular disease	7 (9%)	13 (13%)	13 (13%)
eGFR, mean (SD)	96 (44)	98 (45)	98 (45)
**Acute COVID-19 Illness Characteristics**			
Admission SOFA score, median [IQR]	3.5 [3-6]	3 [2-5]	3 [2-5]
Steroid use, n (%)	39 (51%)	43 (41%)	47 (46%)
IL-6 receptor inhibitor use, n (%)	17 (22%)	26 (25%)	23 (23%)
Oxygen use			
None	0 (0%)	0 (0%)	0 (0%)
Nasal Cannula	23 (30%)	31 (30%)	32 (31%)
Non-Rebreather	17 (22%)	18 (17%)	18 (18%)
NIPPV or HFNO	4 (5%)	2 (2%)	2 (2.0%)
Mechanical ventilation, n(%)	32 (42%)	53 (51%)	50 (49%)
Ventilator days, median (IQR)	31 [12-42]	35 [18-46]	35 [17-46]
Hospital Days, median [IQR]	18 [7-35]	23 [8-47]	23 [7-46]

*Ever smoker: former or active smoker. COPD: chronic obstructive pulmonary disease. eGFR: estimated glomerular filtration rate based upon CKD-EPI equation.[1] SOFA: sequential organ failure assessment. NIPPV: non-invasive positive pressure ventilation. HFNO: high-flow nasal oxygen.

Supplement Table 2. Fibrotic-like abnormalities on thoracic CT scans in the Columbia (discovery), University of British Columbia (validation), and McGill (validation) cohorts.

**Cohort**	**Columbia 4-month follow-up (n=76)**	**Columbia 15-month follow-up (n=104)**	**Columbia 3-year follow-up (n = 102)**	**UBC 3-month follow-up (n = 56)**	**McGill Hospital DC, 4-month, or 15-month follow-up (n=37)**
Months from admission to thoracic CT, median [IQR]	4.5 [4.0-4.8]	15 [14.2-16.5]	39 [37-42]	3.2 [2.4-3.7]	2.6 [0.13-5.7]
Any fibrotic pattern, n (%)	45 (59%)	67 (64%)	62 (62%)	32 (57%)	16 (43%)
Reticulations, n (%)	30 (39%)	67 (64%)	58 (57%)	31 (55%)	15 (41%)
Traction Bronchiectasis, n (%)	21 (28%)	41 (39%)	46 (45%)	28 (50%)	5 (14%)
Honeycombing, n (%)	1 (1.0%)	1 (1.0%)	1 (1.0%)	2 (3.6%)	4 (11%)

Supplement Table 3. Comparison of demographic and clinical characteristics between those with and without fibrotic-like abnormalities in the McGill (validation) nested case-control cohort.

	Case: Fibrotic-Like Abnormalities (n=16)	Control: No Fibrotic-Like Abnormalities (n = 21)	p-value
Age, mean (SD)	67 (10)	63 (18)	0.36
Male, n (%)	12 (75%)	10 (48%)	0.18
Body Mass Index (kg/m2), mean (SD)	28 (4.0)	30 (6.6)	0.34
Race/Ethnicity, n (%)			
White	11 (69%)	13 (62%)	0.72
Asian	2 (13%)	2 (10%)	
Hispanic	2 (13%)	1 (4.8%)	
Middle Eastern	1 (6.2%)	3 (14%)	
Other	0 (0%)	1 (4.8%)	
**Comorbidities**			
Smoking status, n (%)			
Never	9 (56%)	16 (76%)	0.17
Active	0 (0%)	1 (4.8%)	
Ever	7 (44%)	3 (14%)	
Unknown	0 (0%)	1 (4.8%)	
COPD, n (%)	6.2 (1%)	0 (0%)	0.89
Asthma, n (%)	2 (13%)	0 (0%)	0.35
Cardiovascular disease, n (%)	5 (31%)	4 (19%)	0.64
eGFR, mean (SD)	82.3 (24.2)	83.4 (27.6)	0.9
**Acute COVID-19 Illness Characteristics**			
Corticosteroid Use, n (%)	12 (75%)	11 (52%)	0.29
IL-6 receptor inhibitor use, n (%)	1 (6.2%)	0 (0%)	0.89
Oxygen Use, n (%)			0.83
None	4 (25%)	3 (14%)	
Nasal Cannula	10 (62%)	16 (76%)	
NIPPV or HFNO	1 (6.2%)	1 (4.8%)	
Mechanical Ventilation	1 (6.2%)	1 (4.8%)	
Ventilator days, median [IQR]	12 [10-13]	33 [33-33]	0.22
Hospital days, median [IQR]	9 [5.8-20]	6 [4.0-16]	0.43
Days from admission to CT scan, median [IQR]	99 [32-147]	8 [0-425]	0.19

*Ever smoker: former or active smoker. COPD: chronic obstructive pulmonary disease. eGFR: estimated glomerular filtration rate based on CKD-EPI equation.[1] NIPPV: non-invasive positive pressure ventilation. HFNO: high-flow nasal oxygen.

Supplement Figure 2. Longitudinal plots of individual CC16 levels among Columbia (discovery cohort) (A) 4-month participants with subsequent follow-up visits and (B) 15-month participants with subsequent follow-up visits. Stratified by presence versus absence of a fibrotic pattern on CT. Bolded lines represent median values of each group.

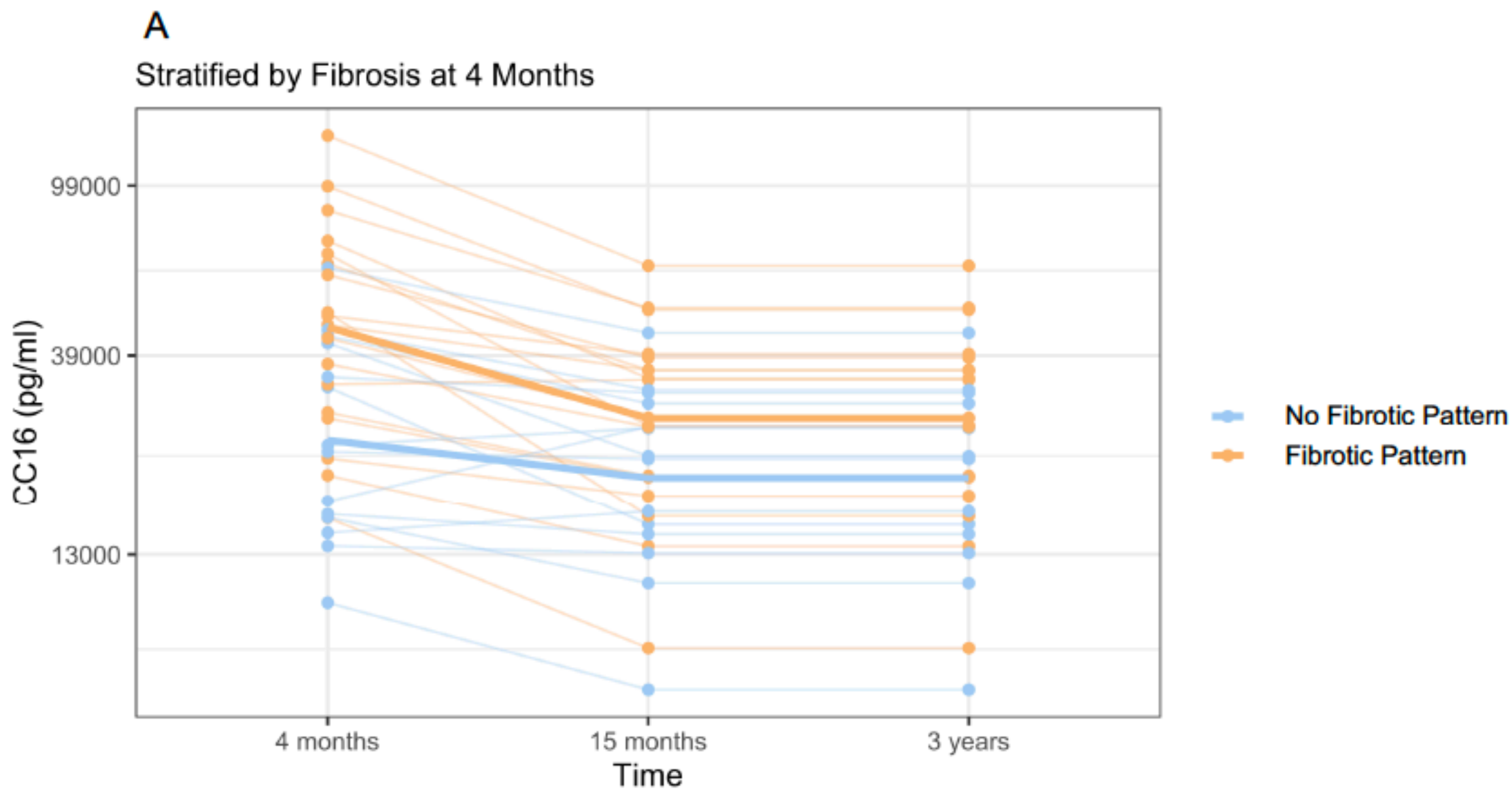

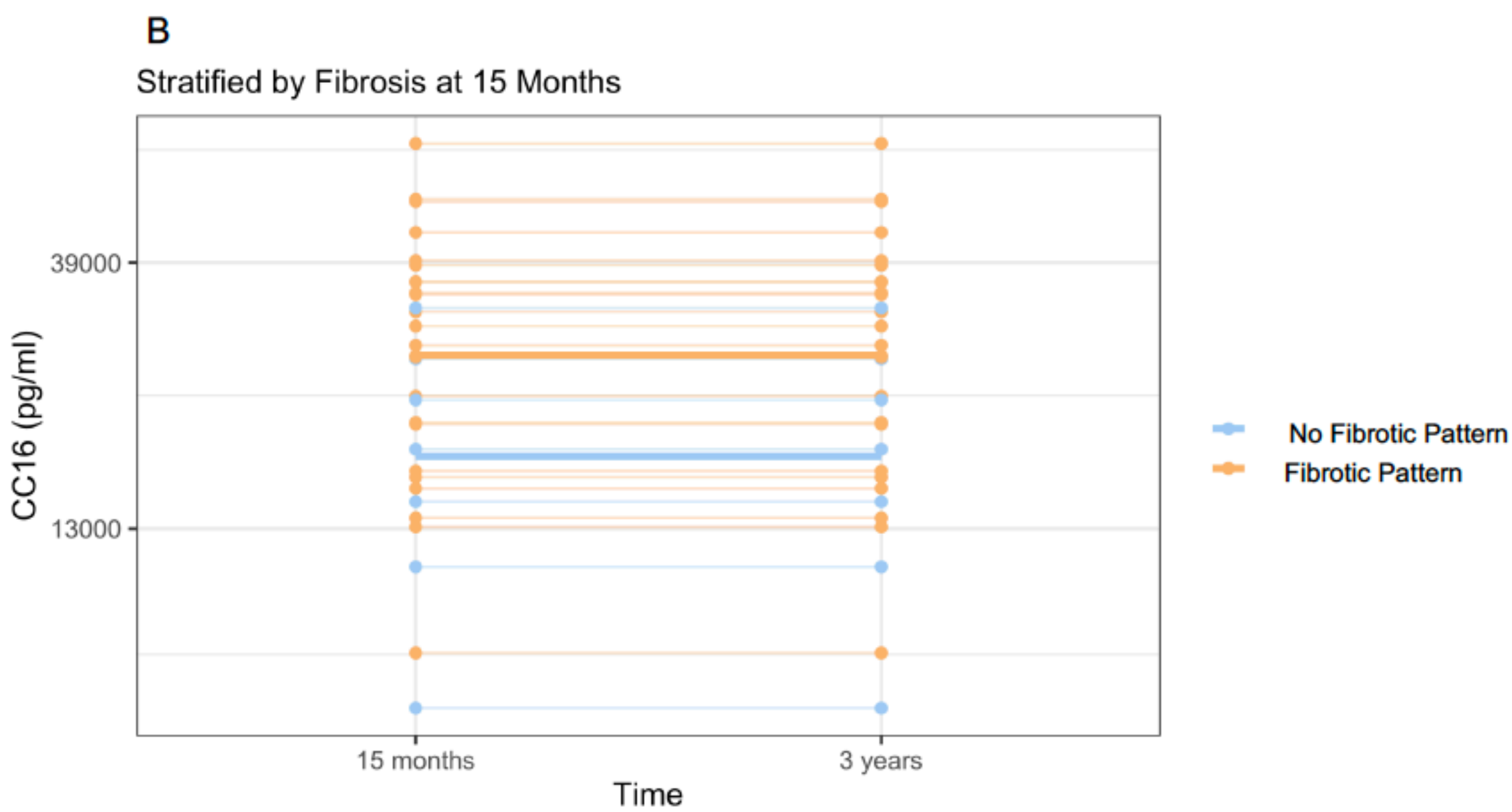

Supplement Table 4. Effect estimates of associations between CC16 and fibrotic-like lung abnormalities on thoracic CT scan in the Columbia (discovery) cohort.

	**CC16 at hospital discharge**		
	**Fibrotic abnormalities at 4 Months**	**Fibrotic abnormalities at 15 Months**	**Fibrotic abnormalities at 3 Years**
Effect per tertile increase in CC16 (pg/ml)	OR (95% CI)	OR (95% CI)	OR (95% CI)
Low	1	1	1
Medium	2.27 (0.47, 13.1)	1.91 (0.56, 7.0)	9.32 (1.97, 56.2)
High	10.0 (2.12, 62.44)	14.3 (2.92, 112)	21.2 (3.76, 188)
p-value for trend	0.007	0.002	0.001
Effect per 1 natural log-fold increase in CC16 (pg/ml)	4.36 (1.43, 17.4)	10.75 (3.02, 55.1)	23.88 (4.20, 280)
p-value	0.02	0.001	0.003
	**CC16 at 4-months**		
	**Fibrotic abnormalities at 4 Months**	**Fibrotic abnormalities at 15 Months**	**Fibrotic abnormalities at 3 Years**
Effect per tertile increase in CC16 (pg/ml)	OR (95% CI)	OR (95% CI)	OR (95% CI)
Low	1	1	1
Medium	2.03 (0.55, 8.08)	5.97 (1.43, 29.3)	3.27 (0.65, 18.7)
High	10.0 (2.62, 45.6)	16.8 (3.20, 137)	21.2 (2.75, 465)
p-value for trend	0.002	0.002	0.007
Effect per 1 natural log-fold increase in CC16 (pg/ml)	7.39 (2.52, 27.4)	17.0 (4.09, 112)	8.64 (2.17, 50.6)
p-value	0.0009	0.0006	0.006
	**CC16 at 15-months**		**CC16 at 3-years**
	**Fibrotic abnormalities at 4 Months**	**Fibrotic abnormalities at 15 Months**	**Fibrotic abnormalities at 3 Years**
Effect per tertile increase in CC16 (pg/ml)	OR (95% CI)	OR (95% CI)	OR (95% CI)
Low	1	1	1
Medium	2.65 (0.90, 8.16)	2.15 (0.63, 7.65)	3.02 (1.12, 8.54)
High	9.38 (2.80, 38.4)	6.83 (1.76, 31.8)	10.19 (3.27, 37.3)
p-value for trend	0.0005	0.008	0.0001
Effect per 1 natural log-fold increase in CC16 (pg/ml)	5.81 (2.10, 19.6)	5.04 (1.55, 20.7)	6.57 (2.67, 19.0)
p-value	0.002	0.014	0.0002

Supplement Table 5. Cross-sectional spearman correlations of CC16 and percent predicted diffusion capacity of lung for carbon monoxide (DLCO) at follow-up in the Columbia (discovery) and University of British Columbia (validation) cohorts.

Columbia	Rho	p-value
4 Months	-0.54	<0.001
15 Months	0.01	0.92
3 years	-0.33	<0.001
UBC		
3 months	-0.39	0.004

Supplement Table 6. Unadjusted and adjusted cross-sectional associations of telomere length and CC16 levels at 4 months, 15 months, and 3 years in the Columbia (discovery cohort).

	**CC16 & Percent Predicted Telomere Length**			**CC16 & Short (<10th percentile) Telomere Length**		
	**4 months**	**15 months**	**3 years**	**4 months**	**15 months**	**3 years**
**Unadjusted**	n = 69	n = 94	n = 102	n = 69	n = 94	n = 102
Spearman's rho (p-value)	-0.3 (0.03)	-0.2 (0.048)	-0.04 (0.6)	0.09 (0.5)	0.1 (0.2)	0.1 (0.2)
**Adjusted GAMs**	n = 67	n = 90	n = 99	n = 67	n = 90	n = 99
p-for-association (p-for-nonlinearity)	0.01 (0.2427)	0.4 (0.08)	0.1 (0.053)	0.2 (0.06)	0.5 (0.1)	0.3 (0.045)
**Adjusted GLMs**	n = 67	n = 90	n = 99	n = 67	n = 90	n = 99
OR (p-value) 95% CI	-12.9 (0.01) -22.6, -3.2	-5.2 (0.2) -13.6, 3.2	0.8 (0.9) -7.6, 9.2	1.6 (0.3) 0.9, 4.8	0.55 (0.5) -1.1, 2.1	0.04 (0.9) -1.3, 1.3

GAM: Generalized additive model with loess smoothers. GLM: Generalized linear logistic regression model. Adjusted for age, sex, COPD, asthma, race/ethnicity, steroid use, tocilizumab use, BMI, pack year history of smoking, ventilator days, visit days, eGFR

Supplement Table 7. Effect estimates of associations between CC16 and fibrotic-like lung abnormalities on chest CT scan in the validation cohorts.

	**University of British Columbia (n = 56)**
	**Adjusted association of CC16 with Fibrotic abnormalities at 3 Months**
Effect per tertile increase in CC16 (pg/ml)	OR (95% CI)
Low	1
Medium	1.89 (0.53 - 7.09)
High	3.56 (0.93 - 15.2)
p-value for trend	0.0763
Effect per 1 natural log-fold increase in CC16 (pg/ml)	2.92 (1.07 - 9.07)
p-value	0.0462
	**McGill University (n = 37)**
	**Adjusted association of CC16 at hospital discharge with fibrotic abnormalities at hospital discharge, 4 months, or 15 months**
Effect per tertile increase in CC16 (pg/ml)	
Low	1
Medium	1.43 (0.24, 9.05)
High	5.41 (0.79, 52.5)
p-value for trend	0.11
Effect per 1 natural log-fold increase in CC16 (pg/ml)	2.36 (0.93, 7.03)
p-value	0.088
	**McGill University Sensitivity Analysis (n = 34)**
	**Adjusted association of CC16 at hospital discharge with fibrotic abnormalities at hospital discharge, 4 months, or 15 months**
Effect per tertile increase in CC16 (pg/ml)	
Low	1
Medium	2.02 (0.24, 19.1)
High	3.37 (0.48, 28.9)
p-value for trend	0.23
Effect per 1 natural log-fold increase in CC16 (pg/ml)	2.11 (0.79, 6.67)
p-value	0.158

Associations are adjusted for age, sex, race/ethnicity, body mass index, smoking history, COPD, asthma, estimated glomerular filtration rate, use of corticosteroids, IL-6 receptor inhibitor therapy, ventilator days during the hospitalization for acute COVID-19, and days between SARS-CoV2 infection and thoracic CT scan.

Supplement Figure 3. Circulating CC16 (pg/ml) levels in those with and without fibrotic-like abnormalities in the McGill (validation) nested case-control cohort, stratified by follow-up time period of thoracic CT scan: (**A**) hospital discharge, (**B**) 4 months; (**C**) 15 months. Sensitivity analysis

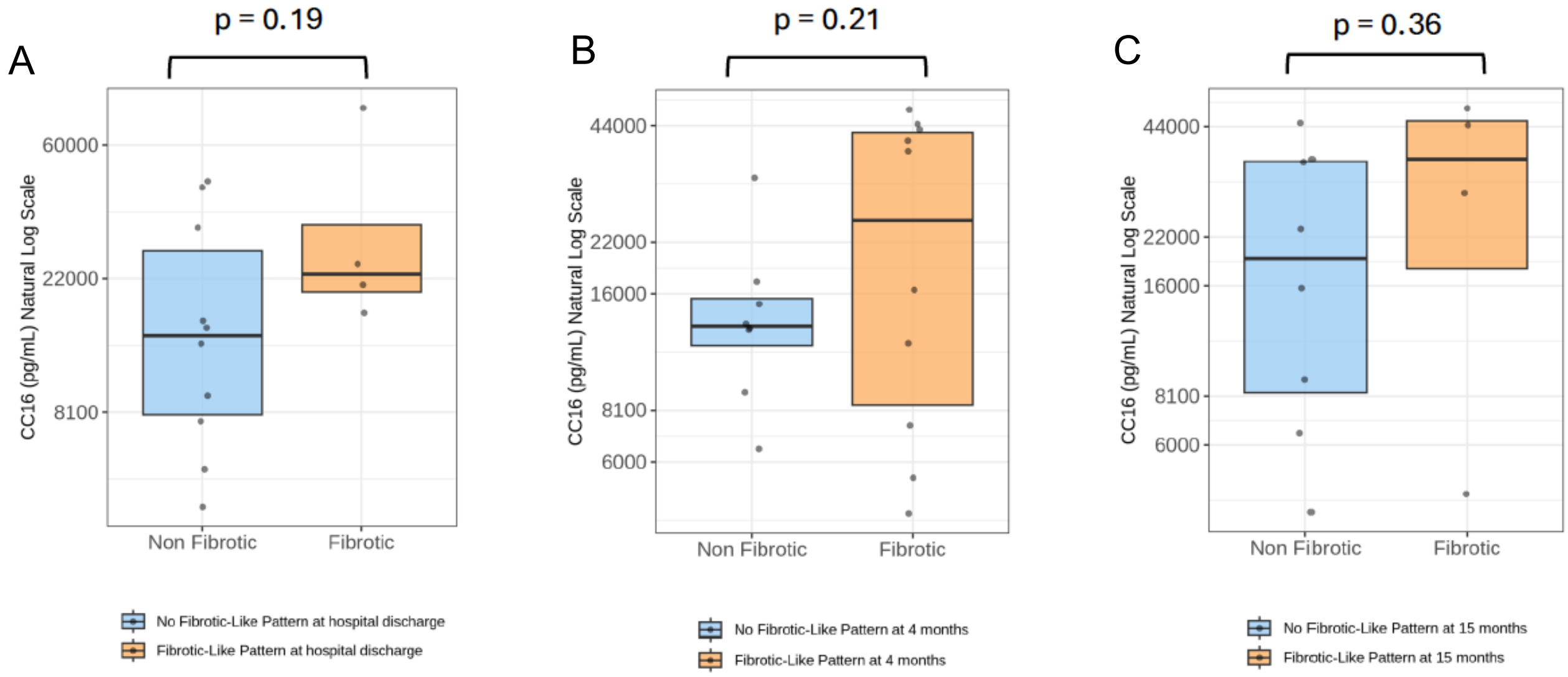

Supplement Figure 4. Circulating CC16 (pg/ml) levels in those with and without fibrotic-like abnormalities in the McGill (validation) nested case-control cohort sensitivity analysis excluding n=3 case patients with honeycombing in the McGill cohort (**A**), and stratified by follow-up time period of thoracic CT scan: (**B**) hospital discharge, (**C**) 4 months; (**D**) 15 months. Sensitivity analysis. (**E**)Generalized additive model (GAM) with LOESS smoothers with adjustment for age, sex, race/ethnicity, BMI, COPD, pack-year history of smoking, estimated glomerular filtration rate, ventilator days, and days since initial SARS-CoV-2 infection using covariate balanced propensity scores.

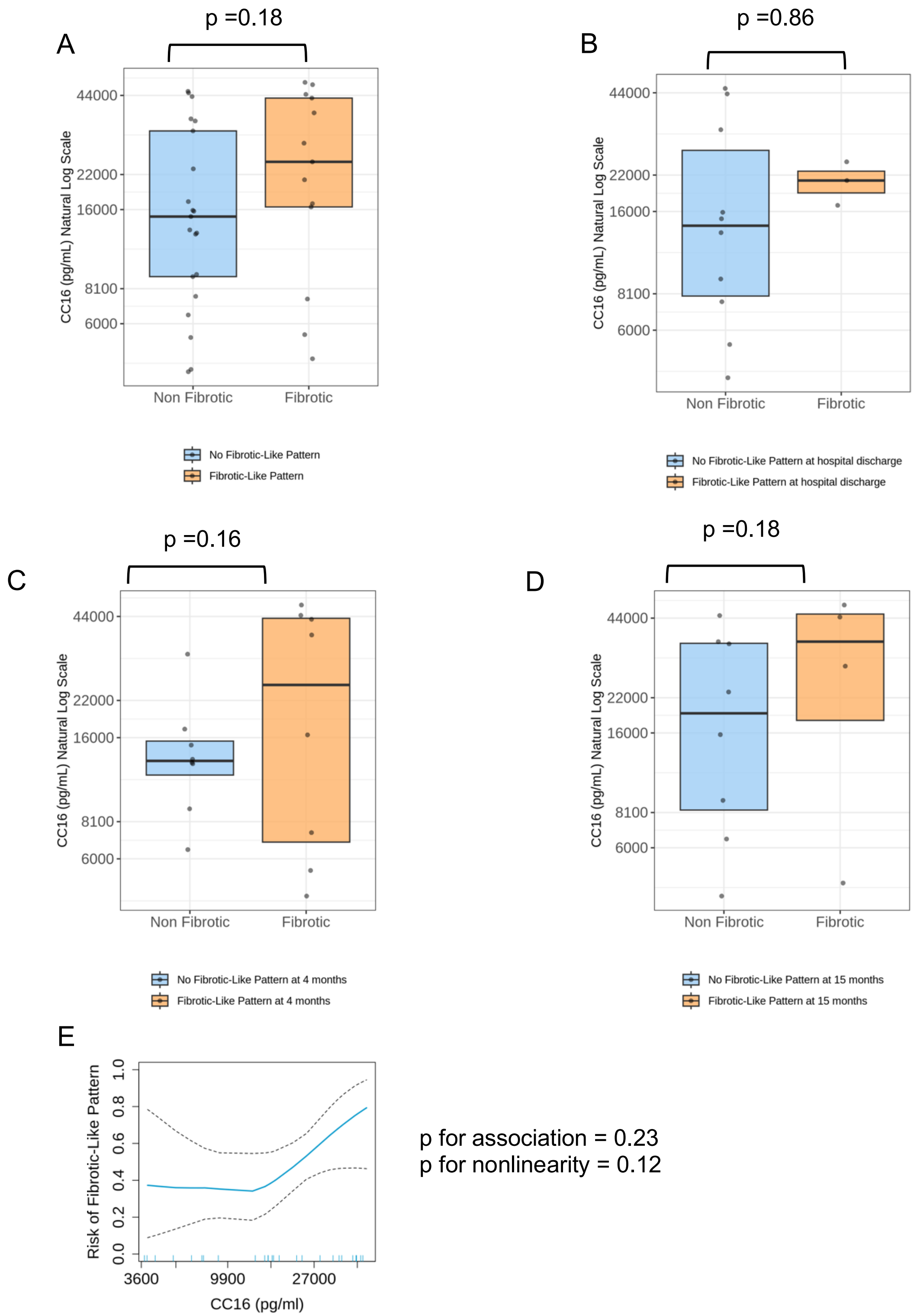

Supplement Table 8. Generalized linear model adjusted associations of CC16 with airway-to-lung ratio 15 months in Columbia (discovery) cohort.

**Association**	**Standard deviation-unit change in airway-to-lung ratio per natural log-fold increase in CC16, β (95% CI)**	**Standard deviation-unit change in airway-to-lung ratio per natural-log fold increase in CC16, β (95% CI)**	**p-value**
Hospital Discharge CC16 - 15-month thoracic CT scan	0.30 (0.10, 0.49)	0.61 (0.21, 1.01)	0.003
4-Month CC16 – 15-month thoracic CT scan	0.29 (0.07, 0.45)	0.58 (0.15, 1.02)	0.01
15-month CC16 – 15-month thoracic CT scan	0.36 (0.18, 0.54)	0.72 (0.36, 1.09)	<0.001

Associations are adjusted for age, sex, race/ethnicity, body mass index, smoking history, COPD, asthma, estimated glomerular filtration rate, use of corticosteroids, IL-6 receptor inhibitor therapy, ventilator days during the hospitalization for acute COVID-19, and days between SARS-CoV2 infection and thoracic CT scan

Supplement Figure 5. Sensitivity analyses of generalized additive model adjusted associations between CC16 and airway to lung ratio. (**A-C**) Complete case analyses among those with all subsegmental airways visualized. (**D-F**) Analyses of all subjects excluded sub-segmental measurements.

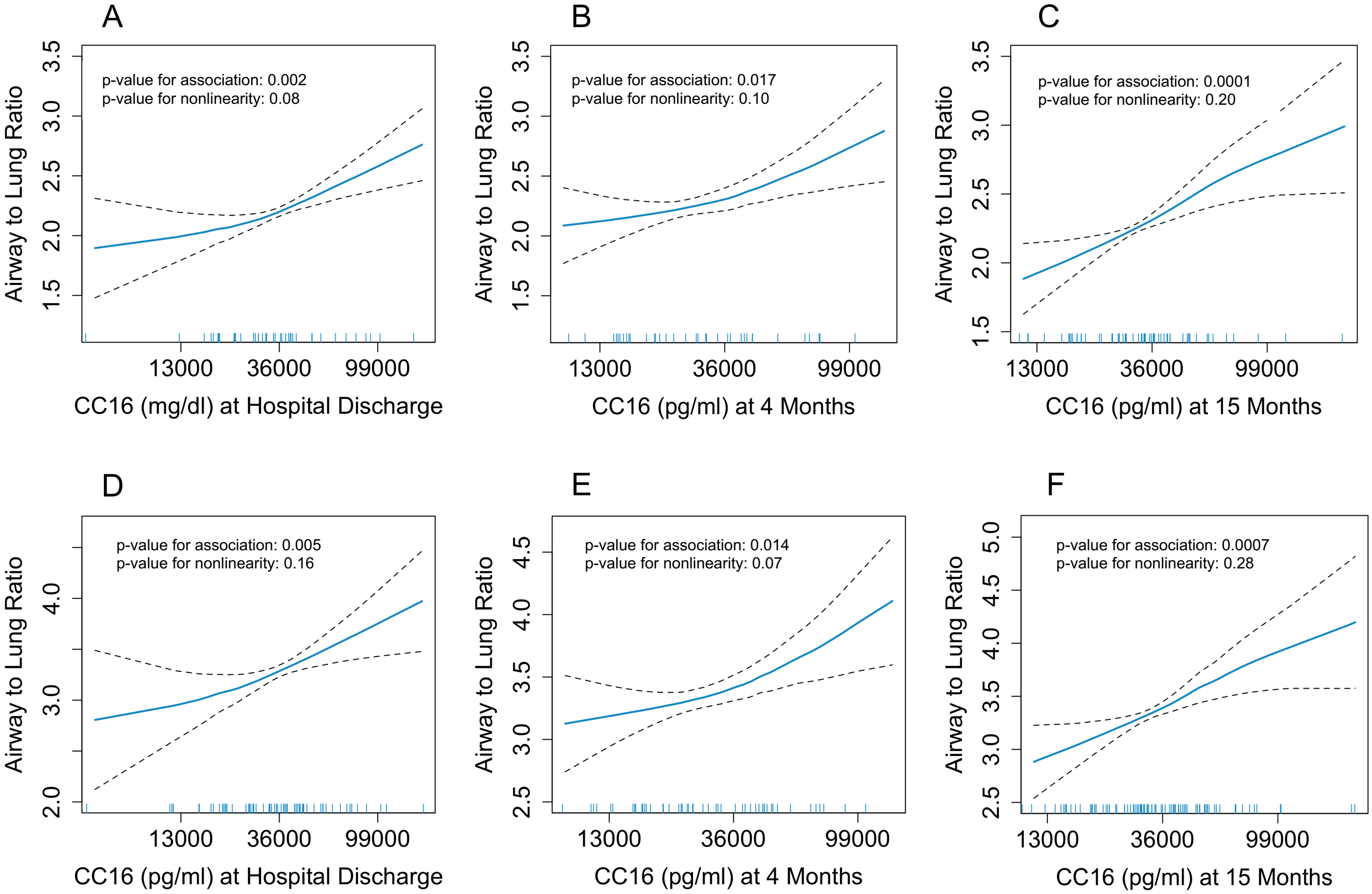

Supplement Figure 6. Histology of lung samples included as controls for the single cell RNA sequencing analysis. Representative hematoxylin and eosin-stained sections of transbronchial lung biopsies from lung transplant recipients undergoing routine surveillance bronchoscopy. There was no evidence of infection or rejection in any of these biopsies. Demographics of these control subjects are described in Table E8.

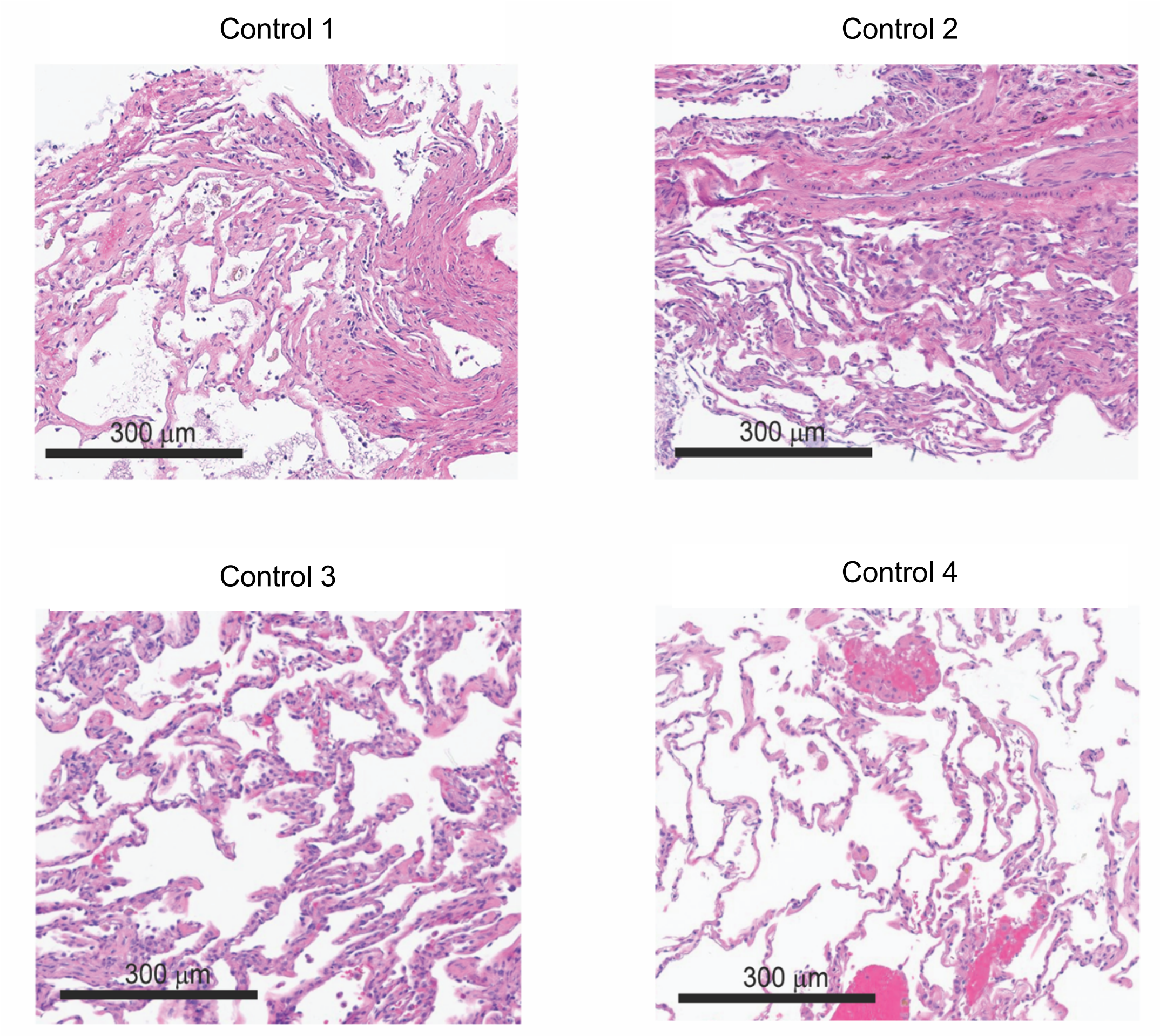

Supplement Table 9. Demographics of COVID-19 pulmonary fibrosis and control patients who underwent bronchoscopy.

Group	Age, years	Sex	Race/Ethnicity	Time in months from COVID-19 infection until bronchscopy	Time in months from lung transplant until bronchoscopy (indication for transplant)
COVID-19	60	Male	Hispanic	50	n/a
COVID-19	36	Male	Hispanic	50	n/a
COVID-19	62	Female	Hispanic	52	n/a
COVID-19	56	Male	Hispanic	51	n/a
Control	39	Female	White	n/a	9 mo (BOS)
Control	66	Female	Asian	n/a	9 mo (non-CF bronch.)
Control	50	Male	White	n/a	3 mo (sarcoidosis)
Control	61	Male	White	n/a	11 mo (COPD/emphysema)

n/a: not applicable. BOS, bronchiolitis obliterans syndrome, non-CF bronch., non-cystic fibrosis bronchiectasis; COPD, chronic obstructive pulmonary disease

Supplement Figure 7. Single cell RNA sequencing quality control, gene expression data, and differences in cell numbers of immune, endothelial, and non-epithelial, non-endothelial, and non-immune cells. A. Proportion of EpCAM+ (blue), CD31+CD45+ (red), and negative (green) cells as determined by expression of *EPCAM*, *PTPRC* (CD45), *PECAM1* (CD31), or none of these genes. One COVID-19 sample which had a low viability and a high proportion of EpCAM-CD31-CD45- cells was not included in the study, leaving a total of four COVID-19 biopsies being used in analyses. Three lung transplant participants had evidence of rejection, and were therefore excluded from the study, leaving a total of 4 lung transplant recipient biopsies being used as controls. A total of eight samples (n = 4 COVID explants and n = 4 lung transplant controls) were included in the single cell RNA sequencing analyses. A total of 41,003 post-COVID cells and 23,516 control cells were included in the analyses. **B**. Feature plot of epithelial cell clusters identified in this study and the genes that characterize each cell cluster **C.** Proportion of different subsets of lung immune and endothelial (CD13+CD45+) cells found in control and COVID-19 samples. **D.** Proportion of different subsets of lung non-epithelial, non-endothelial, and non-immune (EpCAM-CD13-CD45-) cells found in control and COVID-19 samples.

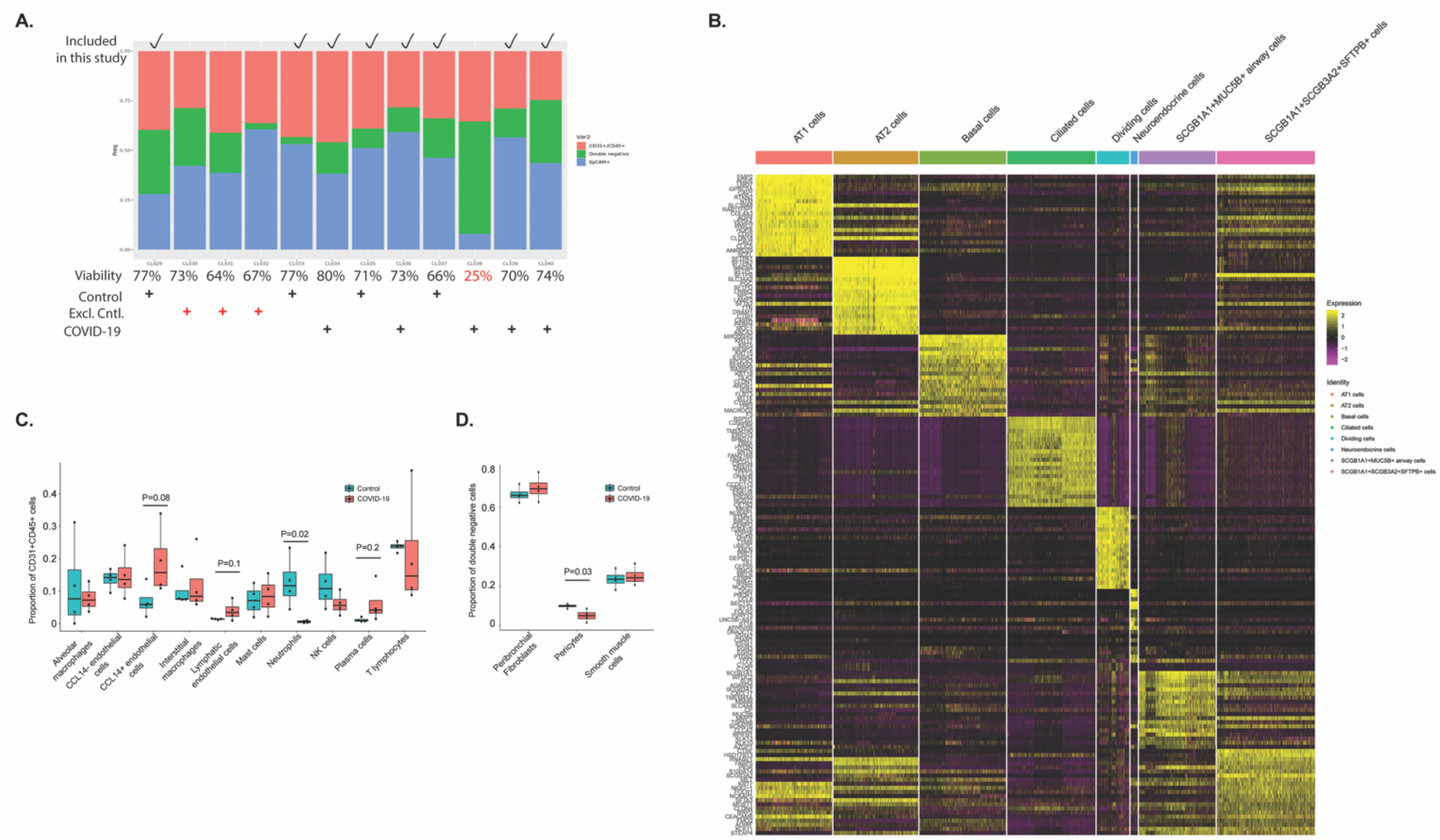

Supplement Table 10. Demographics of patients who underwent lung transplantation for COVID-19 pulmonary fibrosis and controls who underwent surgical resection of pulmonary nodules.

Group	Age, years	Sex	Race/Ethnicity	Time in months from COVID-19 infection until lung transplant	Histopathology findings
COVID-19	61	Male	White	3	Nonspecific interstitial pneumonitis (NSIP) with honeycombing, multinucleated giant cells in airspaces, peribronchial metaplasia, mucostasis, fibroblastic foci and focal ossification
COVID-19	56	Male	White	3	Nonspecific interstitial pneumonitis (NSIP), Diffuse alveolar damage (DAD), multinucleated giant cells in airspaces, peribronchial metaplasia, mucostasis, focal organizing pneumonia, increased macrophages, infarct, and hyaline membranes
COVID-19	64	Male	Hispanic	12	Nonspecific interstitial pneumonitis (NSIP), Diffuse alveolar damage (DAD), peribronchial metaplasia, mucostasis, focal organizing pneumonia, hyaline membranes, and increased macrophages
COVID-19	51	Female	Hispanic	2	Nonspecific interstitial pneumonitis (NSIP), Diffuse alveolar damage (DAD), multinucleated giant cells in airspaces, peribronchial metaplasia, mucostasis, increased macrophages, and organizing pneumonia
COVID-19	48	Female	Unknown	7	Nonspecific interstitial pneumonitis (NSIP), Diffuse alveolar damage (DAD), multinucleated giant cells in airspaces, peribronchial metaplasia, mucostasis, calcification, increased macrophages
COVID-19	63	Female	Hispanic	6	Nonspecific interstitial pneumonitis (NSIP), Diffuse alveolar damage (DAD), multinucleated giant cells in airspaces, peribronchial metaplasia, mucostasis, calcification, increased macrophages, pleural adhesions, organizing pneumonia, and infarct
COVID-19	70	Male	Hispanic	29	Nonspecific interstitial pneumonitis (NSIP), and peribronchial metaplasia,
Control	48	Female	White	n/a	Normal lung tissue adjacent to a granuloma
Control	50	Female	White	n/a	Normal lung tissue adjacent to carcinoid
Control	77	Female	Black	n/a	Normal lung adjacent to NSCLC
Control	52	Female	Black	n/a	Normal lung adjacent to NSCLC
Control	64	Female	Hispanic	n/a	Normal lung adjacent to NSCLC
Control	74	Female	White	n/a	Normal lung adjacent to NSCLC
Control	68	Female	White	n/a	Normal lung adjacent to NSCLC
Control	68	Male	White	n/a	Normal lung adjacent to NSCLC
Control	54	Male	White	n/a	Normal lung adjacent to NSCLC
Control	69	Male	White	n/a	Normal lung adjacent to NSCLC
Control	61	Male	Other	n/a	Normal lung adjacent to NSCLC

n/a: not applicable.

Supplement Figure 8. Immunofluorescence antibody controls. A. Representative photomicrographs of human lung tissue immunofluorescence either without (upper) or with (lower) primary rabbit antibody that recognizes SCGB1A1. The secondary antibody was added to both. The imaging was performed using the channel to recognize the Donkey anti-rabbit secondary antibody covalently linked to an Alexa Fluor Plus 647. Bars represent 100 microns. **B**. Representative photomicrographs of human lung tissue immunofluorescence either without (upper) or with (lower panels) primary antibodies (as described). Secondary antibodies including Donkey anti-rabbit Alexa Fluor Plus 647 and Goat anti-mouse Alexa Fluor 488 were added to all. Imaging was performed using channels to recognize the Donkey anti-rabbit secondary antibody covalently linked to an Alex Fluor Plus 647 (left), or Goat anti-mouse secondary antibody covalently linked to Alexa Fluor 488 (middle), or both (right). Bars represent 50 microns.

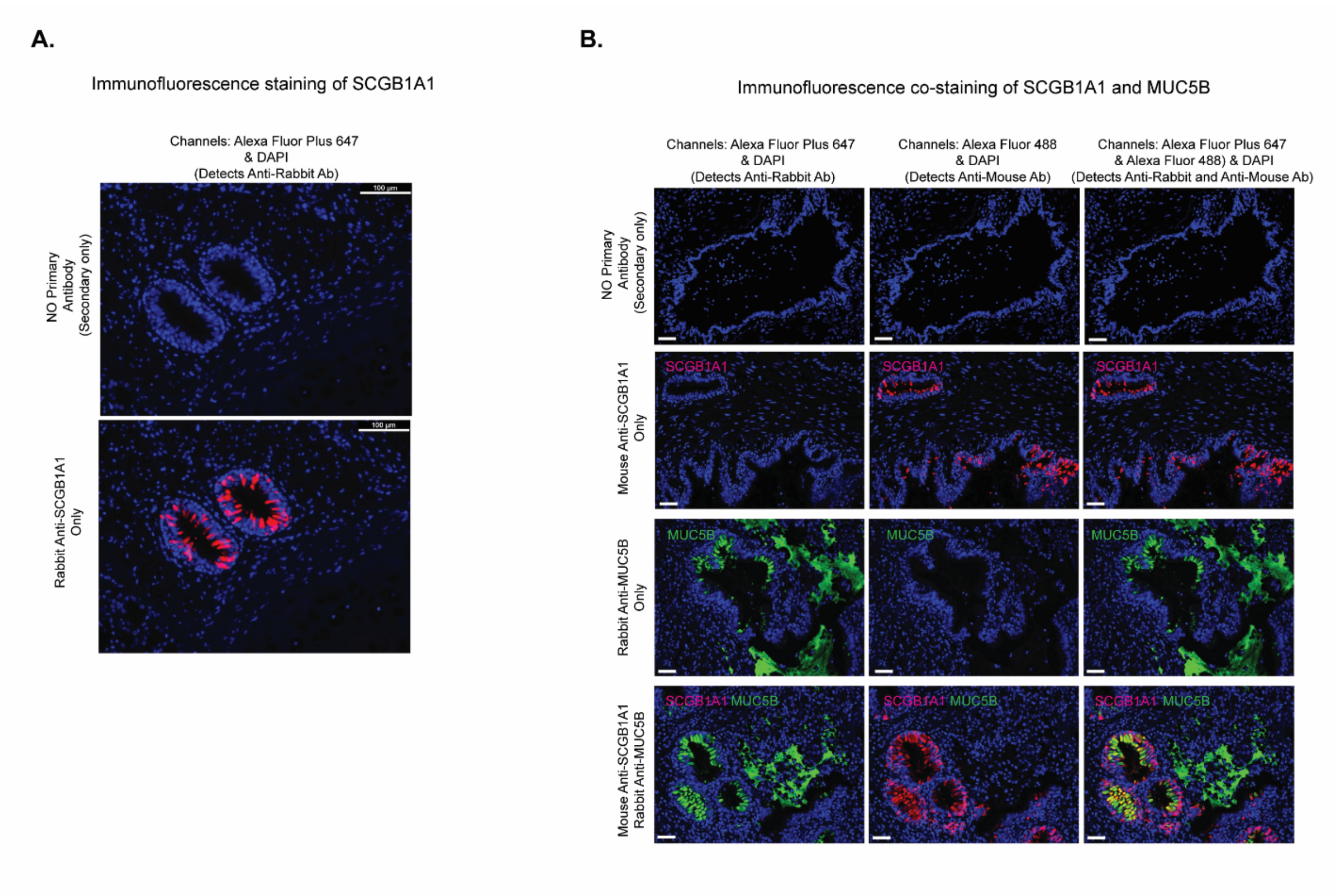

Supplement References

1. Delgado C, Baweja M, Crews DA-O, et al. A Unifying Approach for GFR Estimation: Recommendations of the NKF-ASN Task Force on Reassessing the Inclusion of Race in Diagnosing Kidney Disease. (1533-3450 (Electronic)).
